# Efficacy and tolerability of minocycline in depressive patients with or without treatment-resistant: a meta-analysis of randomized controlled trials

**DOI:** 10.3389/fpsyt.2023.1139273

**Published:** 2023-06-05

**Authors:** Youjia Qiu, Aojie Duan, Ziqian Yin, Minjia Xie, Zhouqing Chen, Xiaoou Sun, Zhong Wang, Xuwei Zhang

**Affiliations:** ^1^Department of Neurosurgery, Lianyungang Hospital of Traditional Chinese Medicine, Lianyungang, Jiangsu, China; ^2^Department of Neurosurgery & Brain and Nerve Research Laboratory, The First Affiliated Hospital of Soochow University, Suzhou, Jiangsu, China; ^3^Suzhou Medical College of Soochow University, Suzhou, Jiangsu, China

**Keywords:** minocycline, depression, treatment-resistant depression (TRD), major depressive disorder (MDD), meta-analysis

## Abstract

**Background:**

Minocycline, an antibiotic with anti-inflammatory, antioxidant, and neuroprotective properties, has been used for treating psychiatric disorders in research. This systematic review aimed to evaluate the efficacy and tolerability of minocycline in patients having depression with or without treatment-resistance.

**Methods:**

Electronic databases including Embase, PubMed, and the Cochrane library were searched for relevant studies published up to October 17, 2022. The primary efficacy outcome was the change in depression severity scores and the secondary efficacy outcomes included the changes in Clinical Global Impression (CGI) and Beck Depression Inventory (BDI) scores and the incidence of response and partial response. Safety outcomes were evaluated based on the incidence of classified adverse events and all-cause discontinuation.

**Results:**

Five studies with 374 patients were selected for analysis. The minocycline group demonstrated a significant reduction in depression severity scale (standardized mean difference [SMD]: −0.59, 95% confidence interval [CI]: −0.98 to −0.20, *P* = 0.003) and CGI (SMD: −0.28, 95% CI: −0.56 to −0.01, *P* = 0.042) scores; however, no statistical difference was found in terms of the BDI score, response, and partial response. No significant differences were found between the groups in terms of adverse events (other than dizziness) and discontinuation rates. Subgroup analysis showed that minocycline was also effective in reducing depression severity scores in treatment-resistant depression (SMD: −0.36, 95% CI: −0.64 to −0.09, *P* = 0.010). Subgroup analysis of Hamilton Depression Rating Scale (17-item) scores showed a statistical difference in response in patients with depression (relative risk: 2.51, 95% CI: 1.13 to 5.57, *P* = 0.024).

**Conclusions:**

Minocycline may improve depressive symptoms and augment response to treatment in patients with depression irrespective of treatment-resistance. However, clinical trials with large sample sizes are warranted for evaluating long-term outcomes with minocycline.

**Systematic review registration:**

https://inplasy.com/inplasy-2022-12-0051/.

## 1. Introduction

Depression is a common chronic mental disorder that contributes to the global burden of disease. Notably, the World Health Organization has recognized it to be the leading cause of disability worldwide (1). Major depressive disorder (MDD) is a form of severe mental dysfunction that affects approximately 6% of adults worldwide and is associated with significant mortality, morbidity, and social costs (2). Several measures, including psychotherapy, physical therapy, and pharmacotherapy, have been adopted for treating MDD. However, approximately 29–46% of patients do not entirely respond to antidepressant therapy (3). In the STAR^*^D trial, the remission rate for MDD was found to be only 36.8% after the first step and 50% after the second step (4).

In this context, patients who do not obtain relief or a satisfactory response after appropriate antidepressant treatment are considered to have treatment-resistant depression (TRD), but there remained discrepancy of the definition of TRD. A recent Delphi-method-based guideline suggested that patients with at least two failed treatments with <25% of improvement when received adequate dosage and duration should be defined as TRD, which reached strong consensus (96%) (5). In addition, although TRD was associated with higher risk of recurrence and poor prognosis, the prevalence of it is hard to estimate (6). In their study, Gaynes et al. found that the TRD-specific hospitalization and average healthcare costs to be higher in these patients than in non-TRD cases (7). Additionally, the depressive symptoms of TRD were associated with unfavorable outcomes. Notably, these patients may demonstrate an increased risk of suicide and functional disability without symptom remission. It is therefore essential that depressive symptoms are relieved in patients with TRD to improve their prognosis.

Notably, conventional antidepressant treatments may lead to persistent residual symptoms and are frequently associated with relapse. The lack of objective biological indicators for the diagnosis of depressive disorders and the limitations of antidepressant treatment have prompted the need to explore new theories and mechanisms for the development of depressive disorders. The Canadian Network for Mood and Anxiety Disorder Treatments guideline currently recommend the use of augmentation therapy (adding another drug that improves the efficacy of an antidepressant). Notably, inflammation has been recognized to be a factor involved in the pathogenesis of MDD (8). Current research on the mechanisms underlying the development of MDD suggests that its progression is associated with activation of immune-inflammatory and oxidative pathways; this indicates that new treatments for depression should focus on attenuation of immune system activation and enhancement of neuroprotective processes (9, 10). In this context, Husain et al. Unlike other antibiotics, it offers the advantage of central nervous system penetration (through the blood-brain barrier); this ability supports its pharmacological use as a neuroprotective agent (11). Notably, the potential neuroprotective and antidepressant effects of minocycline have been confirmed in animal and human studies, and previous meta-analyses have explored clinical outcomes following its use in depression (1, 12, 13). However, the scope of these studies was limited by the omission of varying degrees of depression.

In view of these issues, we performed this systematic review and meta-analysis of randomized controlled trials (RCTs) that evaluated the efficacy and safety of minocycline in depression. Subgroup analyses were performed to evaluate minocycline use in MDD and TRD and clinical responses to minocycline treatment.

## 2. Material and methods

This meta-analysis was performed according to the Preferred Reporting Items for Systematic Reviews and Meta-Analyses Statement (14) and has been registered on the International Platform of Registered Systematic Review and Meta-analysis Protocols (No.: 2022120051).

### 2.1. Search strategy

In order to find relevant studies, a comprehensive literature search was performed across the PubMed, Cochrane, and Embase databases up to October 17, 2022. The details of the search strategy are shown in Supplementary Table S1. The reference lists of the included RCTs, meta-analyses, and reviews were also searched extensively to ensure that the search was comprehensive.

### 2.2. Eligibility criteria

All identified studies were assessed to determine whether they met the Population, Intervention, Comparison, Outcomes and Study criteria. The included studies had the following characteristics: (a) population: adult subjects diagnosed with depression according to the guidelines of the Diagnostic and Statistical Manual for Mental Disorders, fourth edition (DSM-IV); the DSM-IV, text revision; or the DSM, fifth edition. Patients with MDD who did not achieve remission after two or more first-line antidepressant therapies were considered to have TRD (7); (b) intervention: patients who received minocycline were defined as the intervention group; (c) comparison: patients who received placebo treatment were categorized into the comparison group; and (d) outcomes: the primary outcome was the change in severity of depression scores (including those of the Montgomery-Asberg Depression Rating Scale [MADRS] and the 17-item Hamilton Depression Rating Scale [HAMD-17]). The secondary outcomes included changes in Clinical Global Impression (CGI) and Beck's Depression Inventory (BDI) scale scores and the incidence of response or partial response after antidepressant treatment (defined as 50% and 25% reduction from the initial baseline depression severity score, respectively). Safety outcomes included adverse events (AEs) and discontinuation events with minocycline intervention vs. placebo; and (e) study design: only RCTs were included for further analysis. The following publications were excluded: (a) case reports, conference abstracts, unfinished RCTs, and cohort studies; (b) studies not published in English; and (c) studies that were evaluated to be of high risk of bias.

### 2.3. Data extraction

Data extraction was performed by two reviewers (YJQ and AJD). The title and abstract for each study were screened and selected by two independent investigators (YJQ and AJD) and disagreements were resolved by an independent reviewer (ZQC). The obtained data included the study design, authors names, year, essential characteristics of the included patients, exclusion and inclusion criteria, diagnostic criteria for MDD, study duration, outcomes, and sample size.

### 2.4. Statistical methods

The STATA 17.0 software package was used for all statistical analyses. Continuous and dichotomous variables have been presented as standardized mean differences (SMDs) and risk ratios (RRs) with 95% confidence intervals (CIs). Continuous variables having medians and interquartile ranges were transformed to means with standard deviation based on the method described by Hozo et al. (15). Random or fixed effect models were used to pool the data based on the heterogeneity among the included studies. Statistical heterogeneity was evaluated using the Chi-square Q test and *I*^2^ statistics. For the Q test, *P* < 0.10 indicated significant heterogeneity; however, for the *I*^2^ test, <25, 50, to 75%, and more than 75% were considered to indicate low, moderate, and high heterogeneity, respectively (16). In cases of high heterogeneity, sensitivity analysis was performed to detect its source (by eliminating each study in turn); *P* < 0.05 indicated a statistically significant result.

### 2.5. Quality and bias assessment

The bias risk was evaluated using the Cochrane Collaboration tool (17). Bias for these studies was assessed based on the following domains: random sequence generation, allocation concealment, blinding of participants and personnel, blinding of outcome assessment, incomplete outcome data, selective reporting, and other bias. Each item was appraised by determining the risk of bias as low, high, or unclear. The Grading of Recommendations Assessment, Development, and Evaluation scale was used to evaluate the quality of included studies. As the number of pooled studies was <10, our study was assessed to have no publication bias. Discrepancies were resolved by another author who did not participate in the process.

## 3. Results

### 3.1. Search results

**Figure 1:**
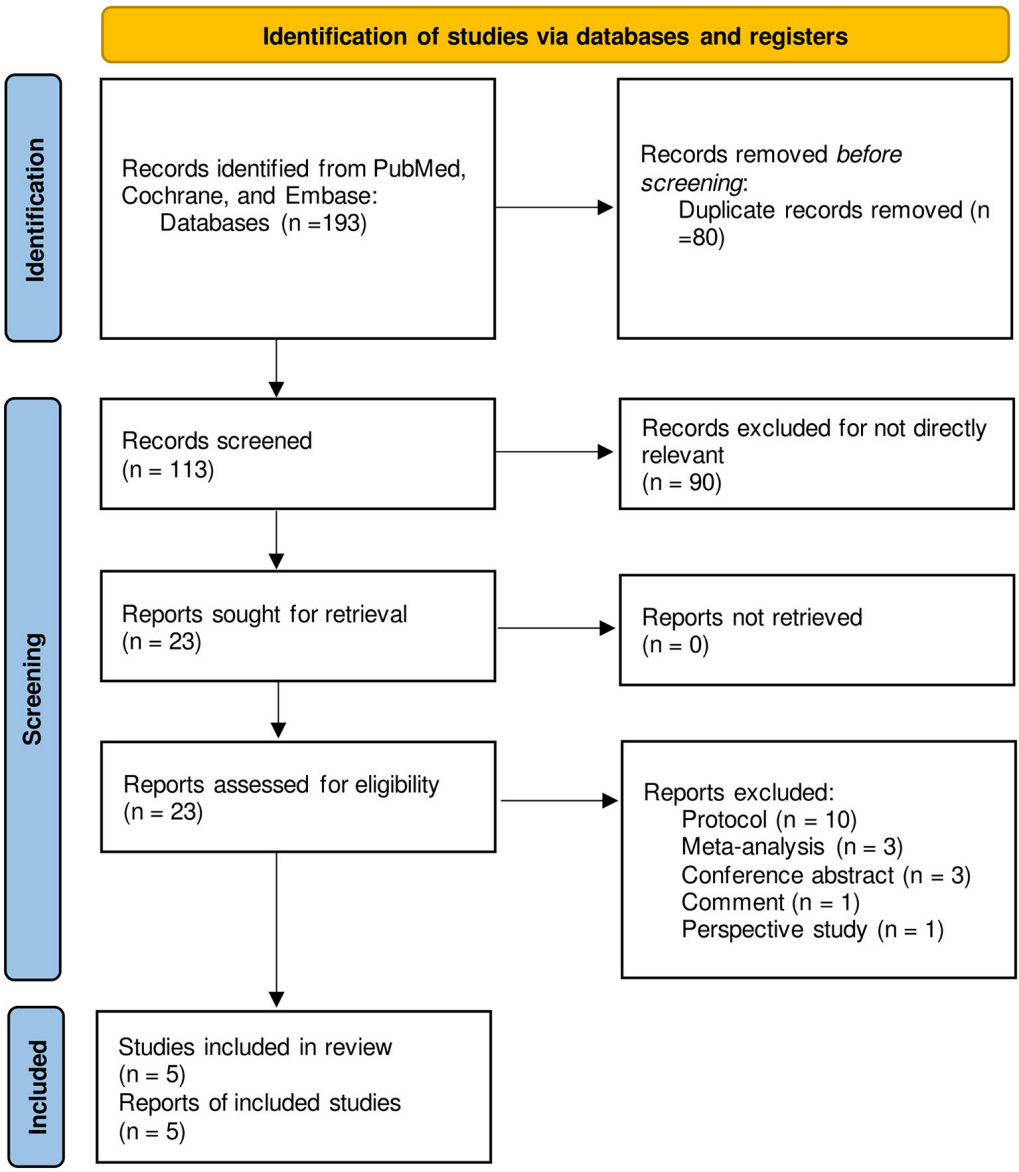
PRISMA flowchart of study identification and selection process.

A total of 193 studies were identified on searching the databases; 80 of them were removed owing to duplication. After scanning the title and abstract of the remaining studies, 90 of them were excluded for irrelevant content. The full texts of 23 studies were reviewed, and 10 protocols, 3 relevant meta-analyses, 3 conference abstracts, 1 comment, and 1 prospective study were excluded; 5 studies were therefore finally selected (18–22). The selection process has been outlined in Figure 1 and the results from each database are shown in Supplementary Table S1.

### 3.2. Study characteristics

A total of 364 participants were enrolled across 5 studies; 178 and 186 were in the minocycline and placebo groups, respectively. Four studies used minocycline as an add-on treatment to standard antidepressants and one used it as monotherapy. Three studies used the DSM, fifth edition for diagnosing depression and one each used DSM-IV and DSM-IV, text revision; one of the studies included human immunodeficiency virus-infected patients with depression. The daily dose of minocycline used in each study was 200 mg, and the duration of administration ranged from 4 to 12 weeks. Table 1 shows the baseline characteristics of the included studies; the inclusion and exclusion criteria for each study are shown in Supplementary Table S2.

**Table 1:** Characteristics and outcome measures of the included trials.

**Included studies**	**Study type**	**Number of participants**	**Mean age (years)**	**Female (%)**	**Treatment durations (weeks)**	**Intervention**	**Outcome measures**	**Depression type**
Husain et al. (20)	DB	40	35	20 (50%)	12	TAU + Minocycline 200 mg/d vs. TAU + Placebo	Score changes in HAMD-17 and CGI. Response (≥50% reduction in HAMD) Quality of life (EQ-5D) Remission (MADRS <9 points)	treatment-resistant depressive symptoms
Dean et al. (22)	DB	71	49.42	47 (66.2%)	12	TAU + Minocycline 200 mg/d vs. TAU + Placebo	Scores changes in MADRS Response (≥50% reduction in HAMD) Quality of life (EQ-5D)	Major depression disorder
Emadi-Kouchak et al. (21)	DB	46	35.53	16 (34.8%)	6	Minocycline 200 mg/d vs. Placebo	Score changes in HAMD-17; Response (≥50% reduction in HAMD); Partial response (25–50% reduction in HAMD)	Mild-to-moderate depression
Nettis et al. (18)	DB	49	45.22	22 (56.4%)	4	TAU + Minocycline 200 mg/d vs. TAU + Placebo	Score changes in HAMD-17, BDI and CGI; Response (50% reduction in HAMD); Partial response (25% reduction in HAMD)	Treatment-resistant depression
Hellmann-Regen et al. (19)	DB	168	46.09	79 (47%)	6	TAU + Minocycline 200 mg/d vs. TAU + Placebo	Score changes in HAMD-6, HAMD-17, MADRS, BDI and CGI Response (50% reduction of MADRS) Remission (MADRS <9 points)	Treatment-Resistant Depression

### 3.3. Primary efficacy outcomes

**Figure 2:**
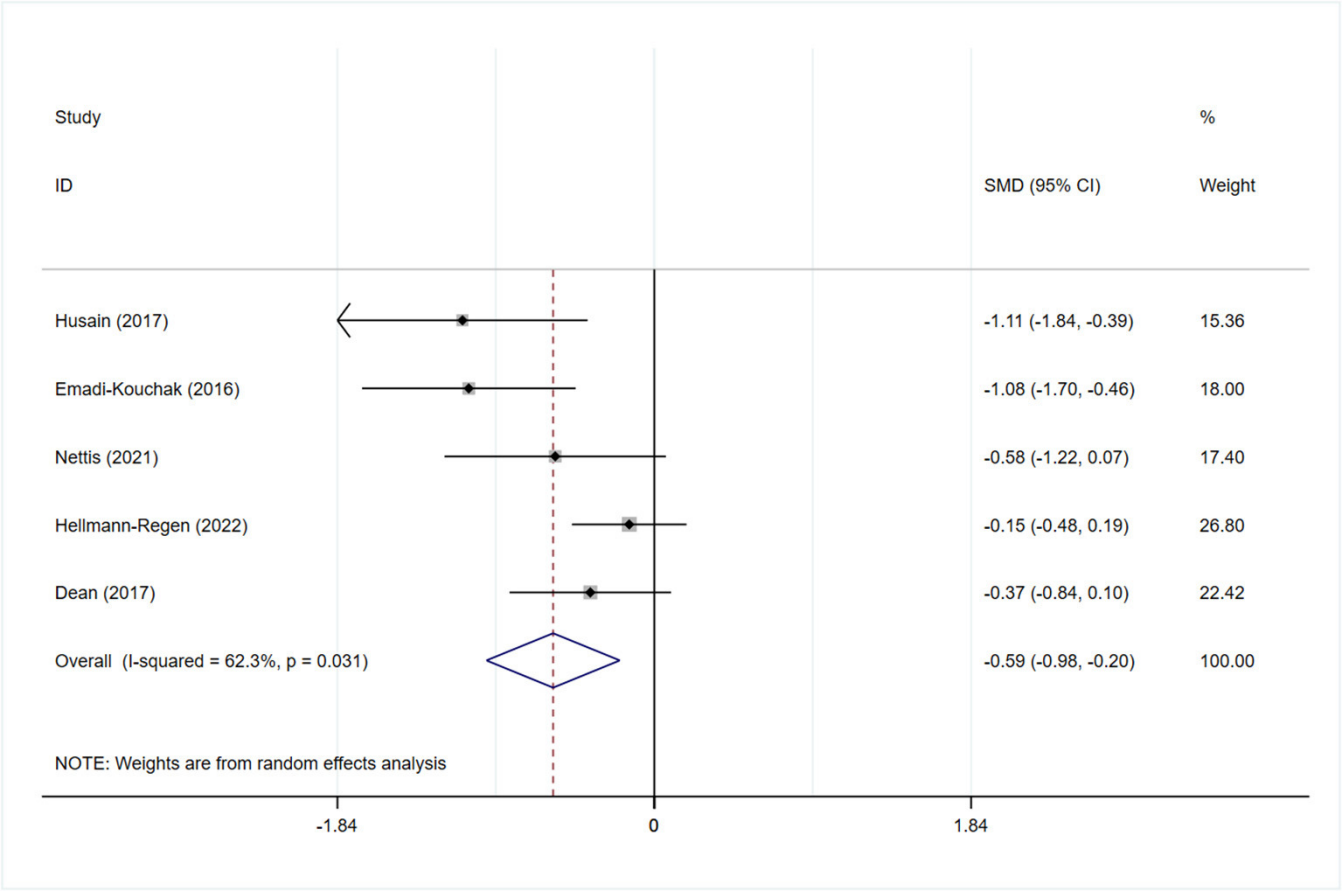
Forest plot for changes in depressive severity score in depressive patients.

**Figure 3:**
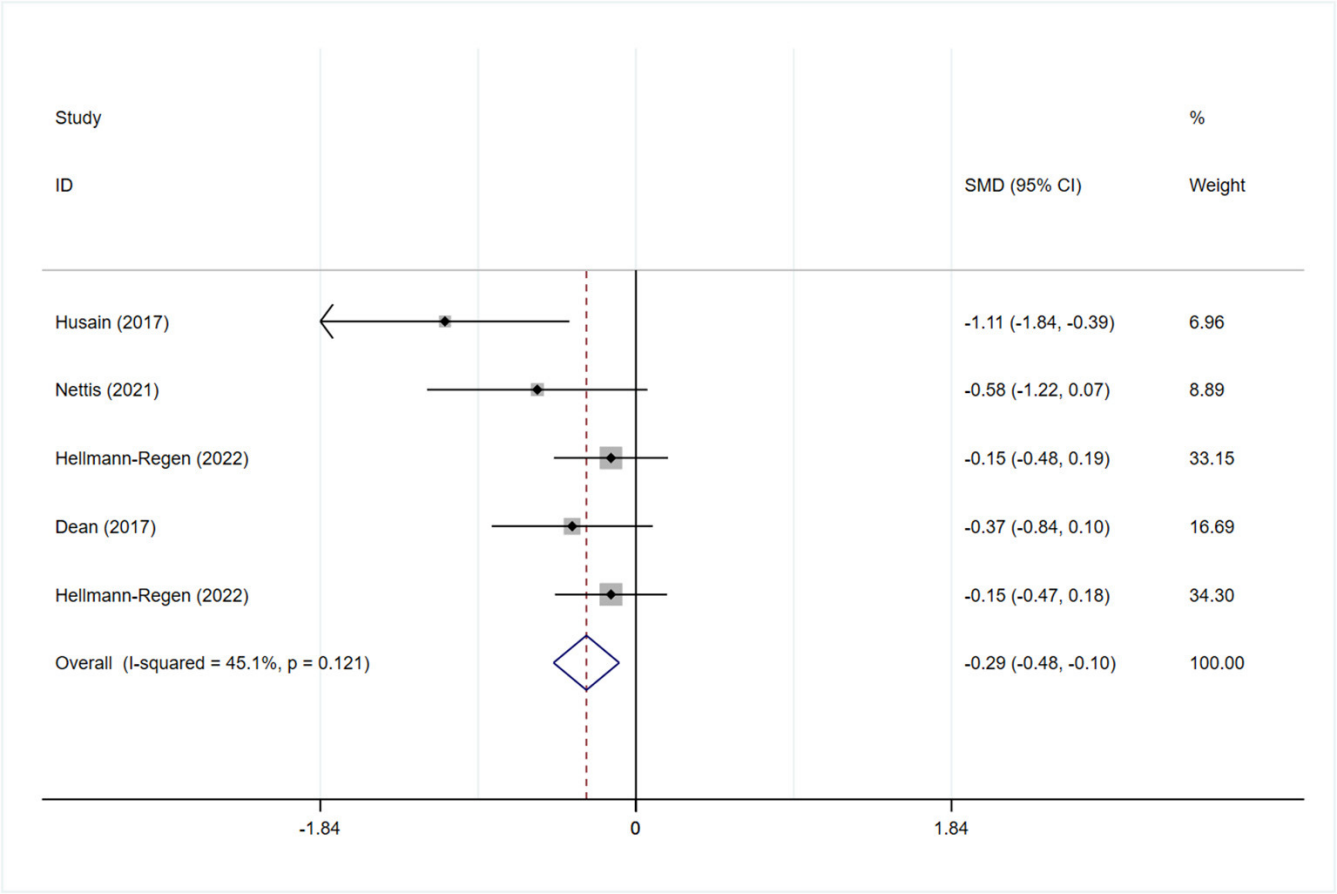
Forest plot for changes in depressive severity score in patients with MDD.

**Figure 4:**
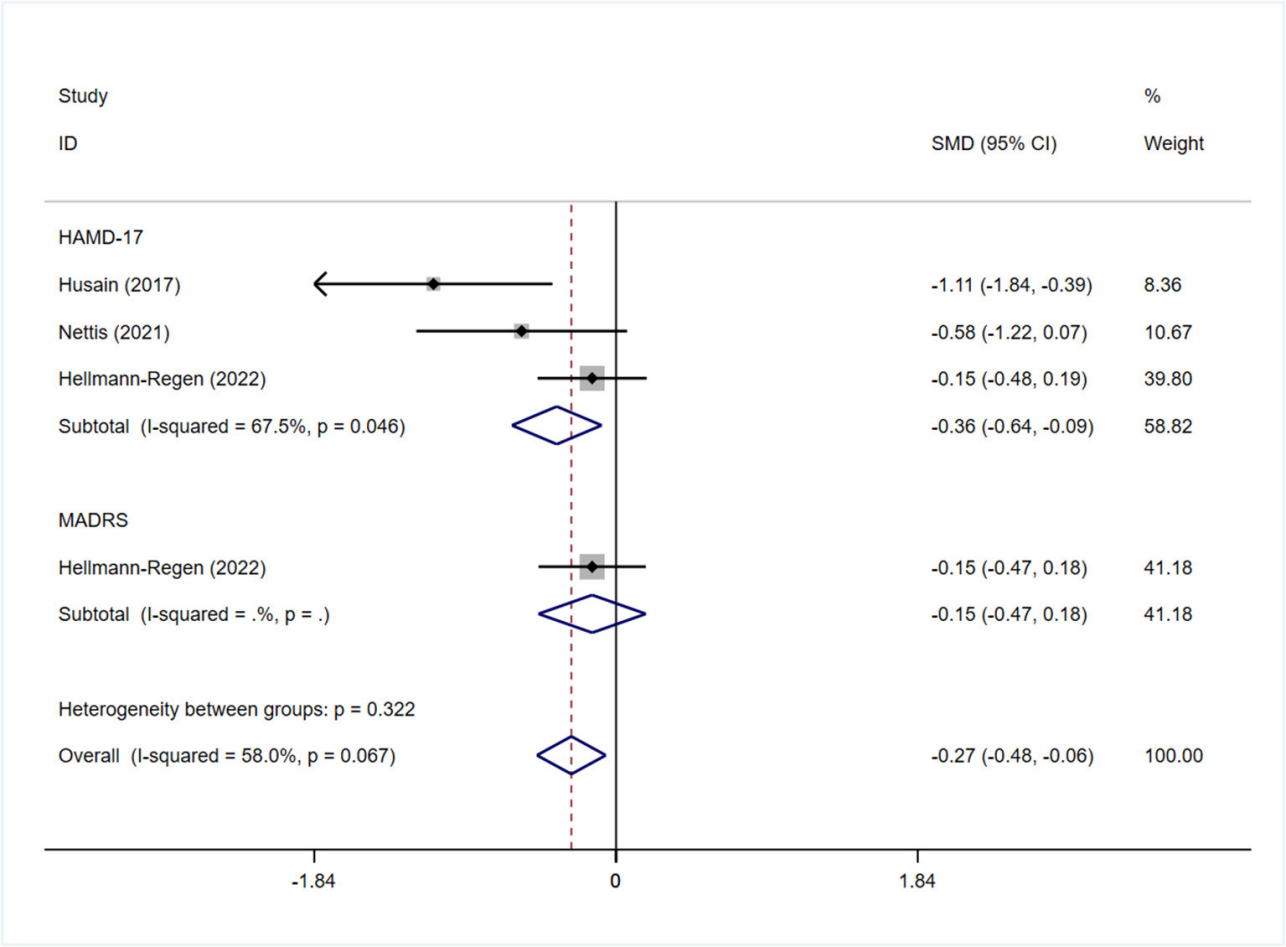
Forest plot for changes in depressive severity score in patients with TRD.

All studies reported the scores of the depression severity assessment scale used for evaluation; four and two studies used the HAMD-17 and MADRS, respectively. In one study that used both of these scales, the scores were separated during subgroup analysis. In patients with depression, the minocycline group demonstrated a greater reduction in depression severity scores (SMD: −0.59, 95% CI: −0.98 to −0.20, *P* = 0.003, *I*^2^ = 62.3%) (Figure 2). During sensitivity analysis, the study Hellmann-Regen et al. was found to be the main source of heterogeneity (19) (Supplementary Figure S1). In MDD, minocycline (as an adjunct to previous medication) showed favorable outcomes (in terms of depression severity scores) compared with placebo (SMD: −0.29, 95% CI: −0.48 to −0.10, *P* = 0.003, *I*^2^ = 45.1%) (Figure 3). Three studies analyzed outcomes in TRD; the results showed statistical significance (SMD: −0.27, 95% CI: −0.48 to −0.06, *P* = 0.038, *I*^2^ = 58%) (Figure 4).

### 3.4. Secondary efficacy outcomes

In terms of secondary outcomes, three studies showed changes in CGI scale scores; they showed that minocycline may offer superior CGI score improvement (SMD: −0.28, 95% CI: −0.56 to −0.01, *P* = 0.042) with high heterogeneity (*I*^2^= 86%) (Supplementary Figure S2). Two studies reported changes in BDI scores after treatment; no statistical difference was observed between the minocycline and placebo groups (SMD: −0.11, 95% CI: −0.41 to 0.18, *P* = 0.456, *I*^2^= 0%) (Supplementary Figure S2). On sensitivity analysis, heterogeneity was mainly derived from the study by Hellmann-Regen et al. (19) (Supplementary Figure S3).

**Figure 5:**
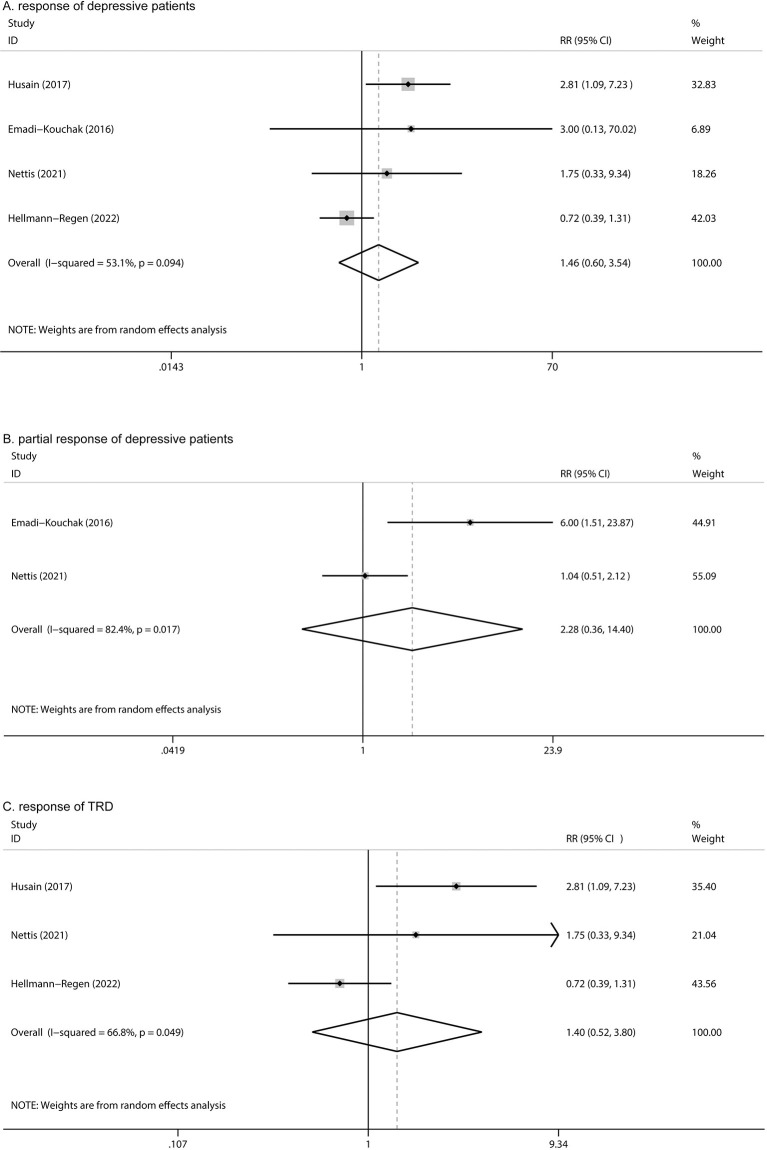
Forest plot for changes in **(A)** response in depression, **(B)** partial response in depression, and **(C)** response of TRD.

Four articles reported responses to minocycline treatment, with no statistically significant difference between the two groups (RR: 1.46, 95% CI: 0.60 to 3.54, *P* = 0.405, I^2^ = 53.1%) (Figure 5A). Two studies reported partial responses (based on 25% reduction in HAMD-17 scores); no significant difference was found between the two groups (RR: 2.28, 95% CI: 0.36 to 14.40, *P* = 0.380) (Figure 5B) and high heterogeneity was observed (*I*^2^= 82.4%). Among the four studies, three reported responses in TRD; no statistically significant difference was found between the two groups (RR: 1.40, 95% CI: 0.52 to 3.80, *P* = 0.506) (Figure 5C).

### 3.5. Safety outcomes

The most reported AEs in the included studies were abdominal pain, asthenia and tiredness, chest palpitation, constipation, flatulence and diarrhea, headache, insomnia, nausea, rash, sore throat, and tinnitus. Except for dizziness, the AEs showed no statistically significant difference in terms of incidence (RR: 2.43, 95% CI: 1.32 to 4.47, *P* = 0.004, *I*^2^= 86%) (Supplementary Figures S4–S7). All included RCTs reported all-cause discontinuation in patients having depression, with no statistically significant differences between the minocycline and placebo groups (RR: 1.44, 95% CI: 0.86 to 2.39, *P* = 0.162) (Supplementary Figure S8).

### 3.6. Subgroup analysis

**Figure 6:**
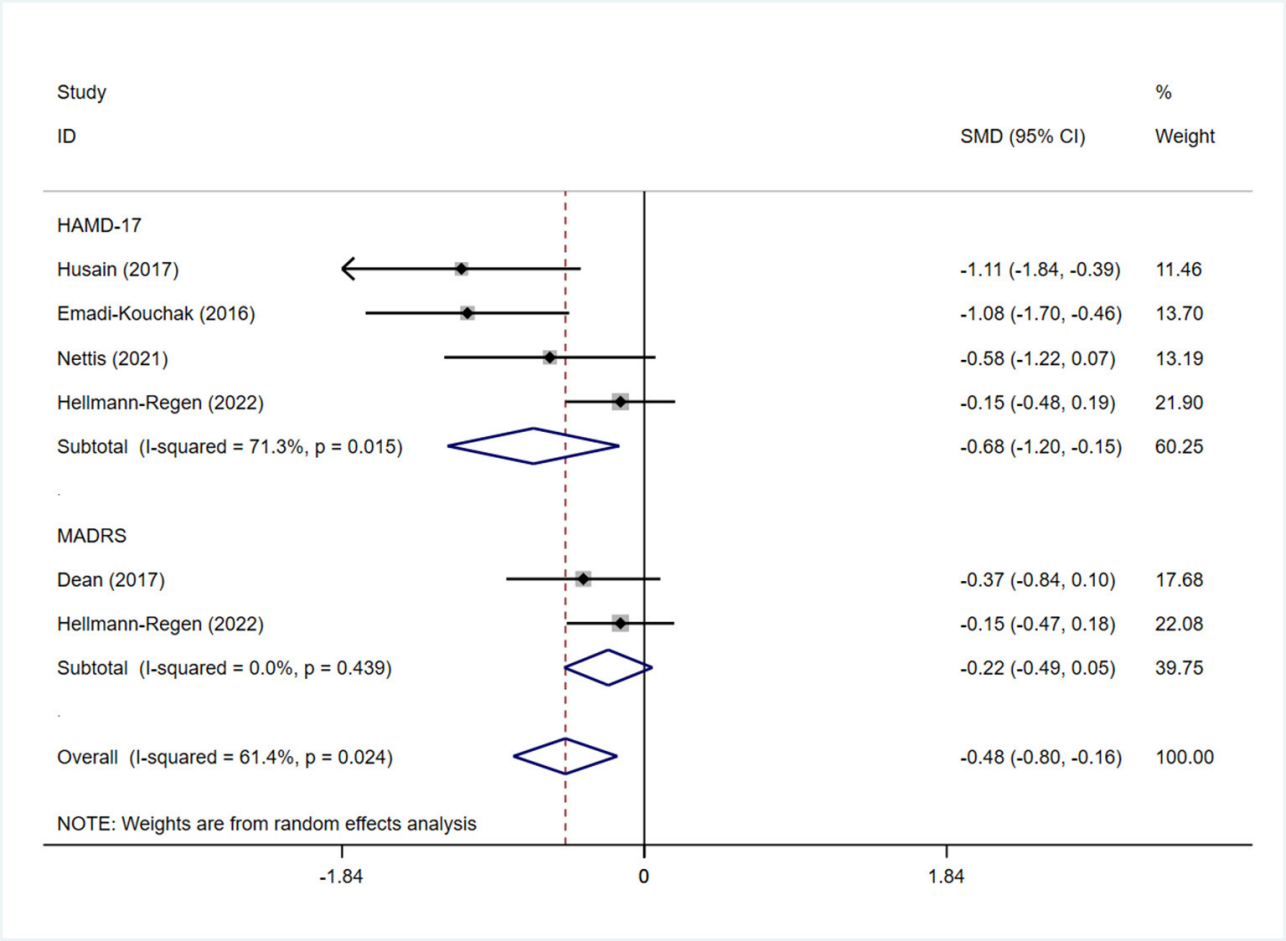
Forest plot for subgroup analysis of HAMD-17 and MADRS in patients with depression.

Subgroup analysis was performed to determine whether the use of different depression-related scales influenced the pooled results of symptom improvement. Patients with depression who received minocycline obtained significant improvement in HAMD-17 scores (SMD: −0.68, 95% CI: −1.20 to −0.15, *P* = 0.011); however, no statistically significant difference was observed in terms of MADRS scores (SMD: −0.22, 95% CI: −0.49 to 0.05, *P* = 0.109) (Figure 6). Four studies reported on changes in the depression severity score in MDD; three of them reported on the outcomes in TRD. The findings in patients with TRD demonstrated a significant difference in HAMD-17 scores between the minocycline and placebo groups (SMD: −0.36, 95% CI: −0.64 to −0.09, *P* = 0.01) (Figure 4). Patients with MDD showed similar changes in depression severity scores, as studies using the HAMD-17 scale focused on patients with treatment-resistant MDD. However, patients with MDD showed no statistically significant difference in terms of MADRS scores (SMD: −0.22, 95% CI: −0.49 to 0.05, *P* = 0.109) (Supplementary Figure S9).

**Figure 7:**
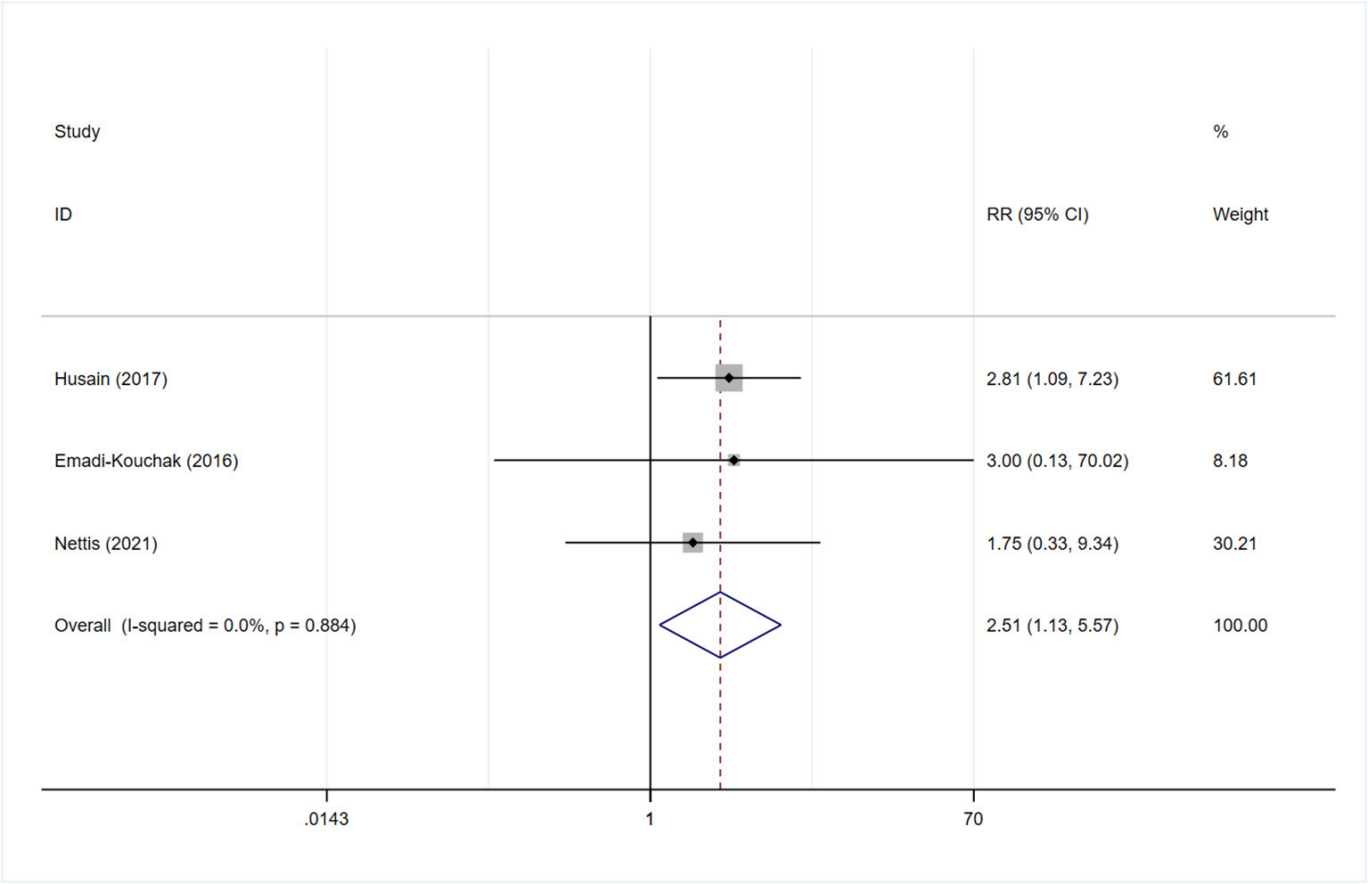
Forest plot for response (defined by 50% reduction of HAMD-17) in depression.

**Figure 8:**
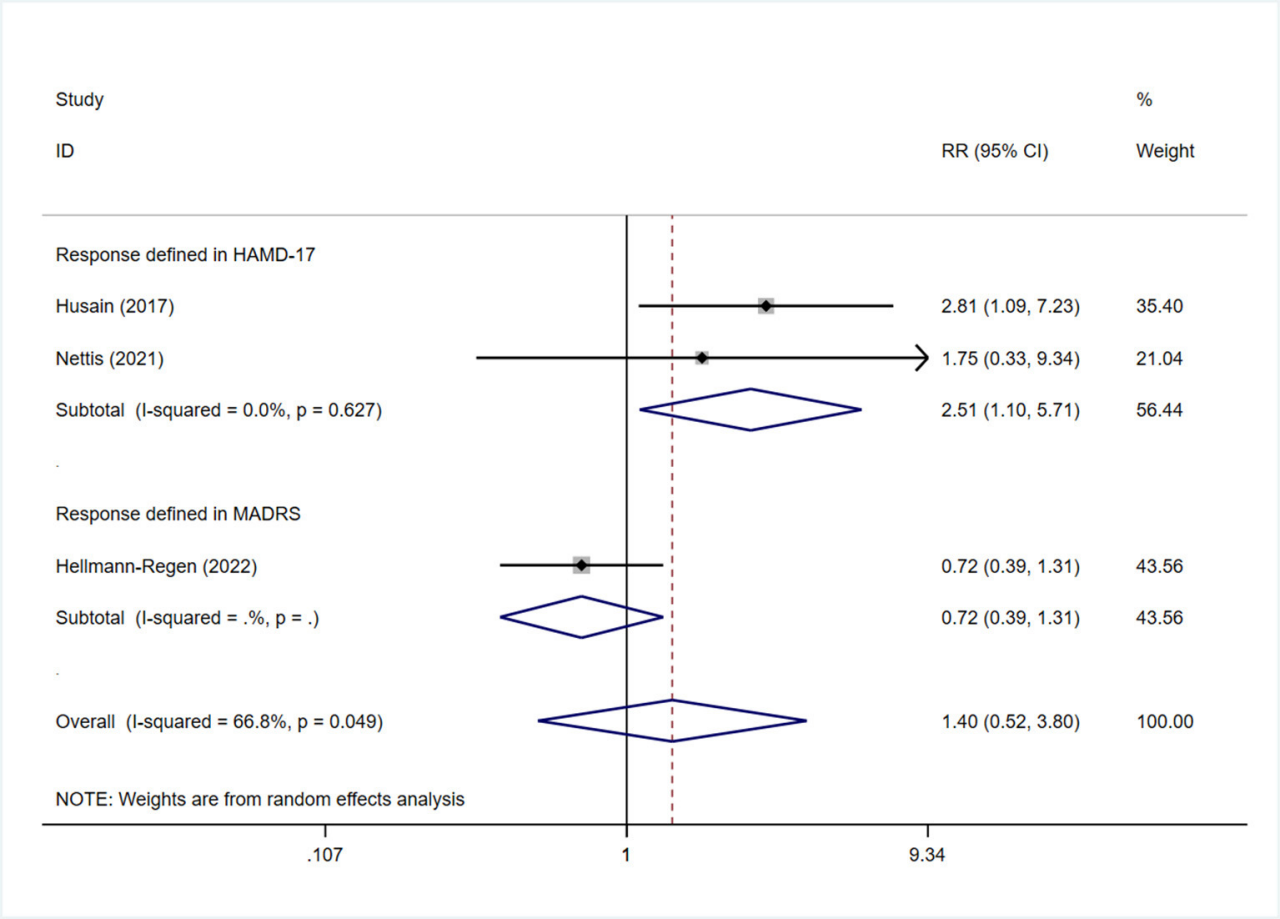
Forest plot for subgroup analysis of response of TRD.

Subgroup analysis was also performed in terms of response, as assessed by the two different scales. Sensitivity analysis showed that the heterogeneity was mainly derived from the study by Hellmann-Regen et al., which used the MADRS scale to evaluate response (Supplementary Figure S10) (19). This study was therefore excluded during evaluation of response based on HAMD-17 scores. The findings showed higher treatment response rates among patients in the minocycline group than in the placebo group (RR: 2.51, 95% CI: 1.13 to 5.57, *P* = 0.024) (Figure 7). Three studies reported on the response to minocycline in TRD; two used HAMD-17 scores for evaluation and one used MADRS scores. The difference in treatment response, as evaluated by HAMD-17 scores, was found to be statistically significant (RR: 2.51, 95% CI: 1.10 to 5.71, *P* = 0.028) (Figure 8), which was in consistent with the outcomes observed in patients with depression.

### 3.7. Quality and risk of bias assessment

The risk of bias across all included RCTs is shown in Supplementary Figures S11, S12; the data indicates that all included RCTs were evaluated to be low-risk in most domains. According to the Grading of Recommendations Assessment, Development and Evaluation scale, the quality of evidence of the included RCTs was relatively high (Supplementary Table S3).

## 4. Discussion

This meta-analysis of RCTs evaluated the efficacy and safety of minocycline as single agent or adjunctive therapy in patients with depression. The finding suggested that the administration of minocycline could reduce HAMD-17 and CGI scores in patients with depression and achieve a higher partial response. On excluding one study using the MADRS scale to evaluate treatment response, the response rate was found to be higher in the minocycline group than in that receiving placebo. In subgroup analysis, it also showed significant reduction of HAMD-17 score in patients with TRD. Moreover, no significant difference was found in between the groups in terms of the incidence of AEs and all-cause discontinuation; this indicates that treatment with minocycline is relatively safe in patients with depression.

The HAMD-17 scale is the most commonly used scoring system in the clinic and has been considered as the gold standard for evaluating depression; however, the MADRS scale is more sensitive to treatment-related changes in depression severity and has been proven to be more effective in large clinical trials (23, 24). As these scales are the most extensively used for assessment of depression severity worldwide, we used both to measure the improvement of symptoms in depressive patients; the minocycline group showed greater reduction in depression severity scores. In this context, Anderson et al. indicated that the degree of depression is a potential marker of antidepressant efficacy in individuals (25). Notably, Fournier et al. also observed that patients who have severe depressive symptoms might respond better to drug intervention than those with mild or moderated symptoms (26). We also did a subgroup analysis on MDD based on their conclusions; the finding suggested that minocycline was also effective than placebo, and achieved greater reduction in depression severity scores. However, we also found that in the study conducted by Husain et al. (27), minocycline demonstrated the optimal efficacy compared with placebo, and there was almost no improvement in HAMD-17 score in the placebo group. The explanation of little response in the placebo group is that the long duration of depression may reduce the placebo response (28). Furthermore, studies have proven that the placebo response may decrease with the increasing baseline depressive severity score, and Fournier also confirmed that this tendency was more significant when the baseline HAMD-17 score was above 25 (26). The baseline HAMD-17 score in study conducted by Husain et al. is 32.6, which is higher than that in other studies (20). In addition, the discrepancy between HAMD-17 and self-reported evaluating scale in placebo group was also observed in their study, which is common and caused by many possible reasons, such as patients' perception and expectancy biases (20, 29).

In our study, subgroup analysis in TRD cases also demonstrated significant improvement in HAMD-17 scores among patients who received minocycline. In view of the discrepancy between scores obtained using different scales, a scale-based subgroup analysis was also performed. The results showed a significant difference in HAMD-17 score. However, this result was mostly influenced by Husain et al. (27). Although the study conducted by Nettis et al. did not demonstrate significance, they found trend levels of significant difference in the reduction of HAMD-17 score and acknowledged the efficacy of minocycline through other assessment methods (18). While in MADRS score, no statistical difference was found in minocycline and placebo in TRD, which might be a setback for adjuvant minocycline treatment in TRD. Therefore, despite meta-analysis demonstrated significant efficacy of minocycline in TRD, it should be cautious to explain this result as the number of studies that demonstrated the significant difference are still limited. In addition, Leucht et al. suggested that the score of one scale corroborate with that of another; in their study, HAMD scores of 10, 20, and 30 approximately corresponded to MADRS scores of 13, 26, and 39, respectively (30). However, in the absence of individual patient data from the included studies, we failed to convert the scores.

Response and remission are the most widely used indicator in studies on depression (31). Unfortunately, these definitions vary across different studies and scales with divergence and contradiction. In this context, response to treatment is defined as a 50% reduction from the initial baseline score. In their study, Leucht et al. (30) evaluated response using different scales and found that 50% reduction using the HAMD scale corresponded with 48% reduction on the MADRS scale; the reduction percentage was also found to be similar. Notably, the authors preferred to use one of the scales to measure the response rates during meta-analysis (30). In view of the discrepancies in the definition and the results of sensitivity analysis, we excluded the study that used the MADRS score to measure response; the findings showed that minocycline offered higher response rates compared with placebo. The heterogeneity was mainly derived from the study by Hellmann-Regen et al. (19). The authors attributed the unfavorable results in the minocycline group to the shorter treatment duration (that could not reflect the true effectiveness of minocycline) (19). The response to minocycline was similar in TRD; this was particularly meaningful, as many antidepressants that are useful in depression lose efficacy in patients with TRD. Adjuvant minocycline may augment the efficacy of standard antidepressant treatment, as it activates anti-inflammatory pathways and has antioxidant properties (32).

The BDI is a self-rating scale that is used to evaluate cognitive and affective impairment in depression. The CGI, another scale used to evaluate improvement in depressive symptoms, assesses both global improvement and severity of illness (31). In our meta-analysis, a significant difference was found between minocycline and placebo groups in terms of change in CGI scores; however, the changes did not significantly differ based on the BDI scores. Notably, previous meta-analyses did not evaluate this difference. In this context, Dean et al. suggested that certain biological mechanisms correspond with improvements in CGI scores and quality of life (22). However, the reason for the discrepancy between global improvement and depressive symptoms remains unclear and needs further investigation.

In this study, no difference was found between the two groups in terms of all-cause discontinuation. There was also no statistical difference between the minocycline and placebo groups in terms of AEs (except for dizziness). This suggests that although antibiotics need to be administered with care in patients without bacterial infections, minocycline is relatively safe for long-term use (33).

Several observational studies have found the circulating levels of inflammatory biomarkers such as interleukin-6, tumor necrosis factor, interferons, and C-reactive protein to be elevated in patients with MDD, especially in those who respond to antidepressant treatment (34). In this context, the degree of peripheral inflammation may affect the pharmacological properties of minocycline as it has anti-inflammatory activity. In their study, Nettis et al. (18) explored baseline levels of peripheral inflammation in responders to minocycline. They concluded that low-grade inflammation (C-reactive protein levels ≥3 mg/L) had an impact on the efficacy of adjuvant minocycline. The levels of interleukin-6 and C-reactive protein may therefore predict minocycline response in patients with depression (18). However, in the *post hoc* stratification of different levels of CRP conducted by Hellmann-Regen et al., no evidence that patients with a higher level of peripheral inflammation had greater response to minocycline was found (19). Therefore, future studies need to identify the association between peripheral inflammation and the efficacy of minocycline.

We conducted the present meta-analysis based on RCTs in contemporary literature to determine the clinical outcomes of minocycline administration in patients with depression. This study evaluated full and partial responses to minocycline treatment and comprehensively assessed symptom improvement in patients with TRD. Current evidence on the administration of adjuvant minocycline in TRD is based on RCTs, and has not been recommended by guidelines. Our meta-analysis may provide a higher level of evidence (35, 36). However, this study has certain limitations. Firstly, the duration of observation with minocycline was variable. Although Hellmann-Regen et al. considered the duration of 6 weeks to be short (19), two other studies having a similar and shorter duration found the outcomes in the minocycline group to be favorable. Nettis et al. also concluded that the antidepressant effect of minocycline was apparent at 4 weeks (18). The optimal duration therefore remains unclear. Secondly, although the dosage was the same across the included studies, the discrepancy between the different medication frequencies was not considered. For instance, in study conducted by Husain et al., patients were required to take minocycline as a single dose and start with 100 mg in the 1^st^ week to encourage compliance, while other studies allowed patients to take minocycline twice a day (27). Whether the discrepancy of different medication frequencies will lead to different improvement in depressive symptoms remains unclear. Thirdly, the long-term outcomes of minocycline administration could not be evaluated due to the short duration of treatment; this needs to be considered in future studies. Lastly, the number of included studies is limited in this meta-analysis, especially in subgroup analysis. Further studies that explore the efficacy of minocycline in MDD and TRD are still needed.

## 5. Conclusion

As an affordable, generic, readily available with low propensity for including antibiotic resistance, minocycline is relatively effective and safe. It can be used widely in patients with depression. Although meta-analysis confirmed that minocycline group had significant improvement in depressive symptom of TRD, the studies are still needed to detect the real-word efficacy of it in TRD. Moreover, future studies need to focus on the long-term efficacy and optimal frequency of minocycline administration. On top of that, large RCTs dealing with the association between inflammation level and minocycline are warranted in the future.

## Data availability statement

The original contributions presented in the study are included in the article/Supplementary material, further inquiries can be directed to the corresponding authors.

## Author contributions

YQ and AD were the principal investigators and drafted the manuscript. XS, ZW, and XZ designed the study and developed the analysis plan. ZC analyzed the data and performed the meta-analysis. ZY and MX revised the manuscript. All authors read and approved the final submitted paper.
