# Supplementary material for: Efficacy and tolerability of minocycline in depressive patients with or without treatment-resistant: a meta-analysis of randomized controlled trials
Source: Front Psychiatry. 2023 Jun 5;14:1139273. doi: 10.3389/fpsyt.2023.1139273 (PMC10277685; doi:10.3389/fpsyt.2023.1139273)
Supplement: Supplementary file 1 [file Data_Sheet_1.docx]

**Efficacy and Tolerability of Minocycline in Depressive Patients with or without Treatment-Resistant: A Meta-analysis of Randomized Controlled trials**

**Youjia Qiu^1,2 #^, Aojie Duan^2, #^, Ziqian Yin^3^, Minjia Xie^2^, Xiaoou Sun^2, *^, Zhong Wang^1,2^, Xunwei Zhang^1, *^**

*^1^* *Department of Neurosurgery, Lianyungang Hospital of Traditional Chinese Medicine, Lianyungang, Jiangsu Province, 222000, China*

*^2^**Department of Neurosurgery & Brain and Nerve Research Laboratory,* *The First Affiliated Hospital of Soochow University, Suzhou, Jiangsu Province, 215006, China*

*^3^ Suzhou Medical College of Soochow University, Suzhou, Jiangsu Province, 215002, China*

^#^ Youjia Qiu and Aojie Duan contribute equally to this work.

* **Correspondence**: Xunwei Zhang, Department of Neurosurgery, Lianyungang Hospital of Traditional Chinese Medicine, Lianyungang, Jiangsu Province, 222000, China, or Xiaoou Sun, Department of Neurosurgery & Brain and Nerve Research Laboratory, The First Affiliated Hospital of Soochow University, Suzhou, Jiangsu Province, 215006, China.

E-mail: zhangxunwei6@163.com or sunxo76@163.com

1. Table S1. Search result of included electronic database
2. Table S2. Inclusion, exclusion criteria, and outcome assessments of the included studies
3. Table S3: Risk of quality assessment (based on GRADE scale)
4. Figure S1. Sensitivity analysis for changes in depressive severity score
5. Figure S2: Forest plot for changes in (A) CGI and (B) BDI score
6. Figure S3. Sensitivity analysis for change of CGI score
7. Figure S4. Forest plot for (A) abdominal pain; (B) asthenia and tiredness; (C) flatulence and diarrhea
8. Figure S5. Forest plot for (A) headache; (B) insomnia; (C) nausea
9. Figure S6. Forest plot for (A) chest palpitation; (B) constipation; (C) sore throat
10. Figure S7. Forest plot for (A) rush; (B) dizziness; (C) tinnitus
11. Figure S8. Forest plot for all-cause discontinuation
12. Figure S9. Forest plot for subgroup analysis of (A) HAMD-17 and (B) MADRS in patients with MDD
13. Figure S10. Sensitivity analysis for response
14. Figure S11: The overall risk of bias
15. Figure S12: The individual risk of bias for each study

**Table S1: Detailed Search Strategy**

Search Date: 17 October 2022

**Pubmed：**

| Search | Query | Results |
| --- | --- | --- |
| #1 | " Depression "[ MeSH Terms] | 249,865 |
| #2 | (Depressive Symptoms[Title/Abstract]) OR (Depressive Symptom[Title/Abstract]) OR (Symptom, Depressive[Title/Abstract]) OR (Emotional Depression[Title/Abstract]) OR (Depression, Emotional[Title/Abstract]) OR (Depressive Disorders[Title/Abstract]) OR (Disorder, Depressive[Title/Abstract]) OR (Depressive Neuroses[Title/Abstract]) OR (Depressive Neurosis[Title/Abstract]) OR (Depression, Endogenous[Title/Abstract]) OR (Depressive Syndrome[Title/Abstract]) OR (Syndrome, Depressive[Title/Abstract]) OR (Neurotic Depression[Title/Abstract]) OR (Unipolar Depression[Title/Abstract]) OR (Depression, Unipolar[Title/Abstract]) | 77,501 |
| #3 | #1 OR #2 | 276,764 |
| #4 | " Minocycline "[ MeSH Terms] | 6,413 |
| #5 | (Minocycline Hydrochloride [Title/Abstract]) OR (Hydrochloride, Minocycline [Title/Abstract]) OR (Minocycline Monohydrochloride [Title/Abstract]) OR (Apo-Minocycline [Title/Abstract]) OR (Apo Minocycline [Title/Abstract]) | 325 |
| #6 | #4 OR #5 | 6,512 |
| #10 | #3 AND #6 | 71 |

**Embase:**

| Search | Query | Results |
| --- | --- | --- |
| #1 | 'depression':ab,ti | 549234 |
| #2 | 'Depressive Symptoms':ab,ti | 79542 |
| #3 | ' Depressive Symptom':ab,ti | 4364 |
| #4 | 'Symptom, Depressive':ab,ti | 9 |
| #5 | ' Emotional Depression ':ab,ti | 69 |
| #6 | ' Depression, Emotional':ab,ti | 301 |
| #7 | ' Depressive Disorders':ab,ti | 15665 |
| #8 | ' Disorder, Depressive':ab,ti | 442 |
| #9 | ' Depressive Neuroses':ab,ti | 46 |
| #10 | ' Depressive Neurosis':ab,ti | 199 |
| #11 | ' Depression, Endogenous ':ab,ti | 23 |
| #12 | 'Depressive Syndrome':ab,ti | 1726 |
| #13 | 'Syndrome, Depressive':ab,ti | 44 |
| #14 | ' Neurotic Depression':ab,ti | 476 |
| #15 | ' Unipolar Depression':ab,ti | 4099 |
| #16 | ' Depression, Unipolar':ab,ti | 87 |
| #17 | #1 OR #2 OR #3 OR #4 OR #5 OR #6 OR #7 OR #8 OR #9 OR #10 OR #11 OR #12 OR #13 OR #15 OR #16 | 581565 |
| #18 | ' Minocycline':ab,ti | 10435 |
| #19 | 'Minocycline Hydrochloride':ab,ti | 415 |
| #20 | ' Hydrochloride, Minocycline':ab,ti | 6 |
| #21 | ' Minocycline Monohydrochloride ':ab,ti | 0 |
| #22 | 'Apo-Minocycline ':ab,ti | 1 |
| #23 | 'Apo Minocycline ':ab,ti | 1 |
| #24 | #18 OR #19 OR #20 OR #21 OR #22 OR #23 | 10435 |
| #25 | #17 AND #24 | 70 |

**Cochrane:**

| Search | Query | Results |
| --- | --- | --- |
| #1 | 'depression': ti,ab,kw | 93851 |
| #2 | 'Depressive Symptoms': ti,ab,kw | 18798 |
| #3 | ' Depressive Symptom': ti,ab,kw | 4980 |
| #4 | 'Symptom, Depressive': ti,ab,kw | 4980 |
| #5 | ' Emotional Depression ': ti,ab,kw | 7851 |
| #6 | ' Depression, Emotional': ti,ab,kw | 197 |
| #7 | ' Depressive Disorders': ti,ab,kw | 11128 |
| #8 | ' Disorder, Depressive': ti,ab,kw | 22730 |
| #9 | ' Depressive Neuroses': ti,ab,kw | 47 |
| #10 | ' Depressive Neurosis': ti,ab,kw | 401 |
| #11 | ' Depression, Endogenous ': ti,ab,kw | 2230 |
| #12 | 'Depressive Syndrome': ti,ab,kw | 2421 |
| #13 | 'Syndrome, Depressive': ti,ab,kw | 2421 |
| #14 | ' Neurotic Depression': ti,ab,kw | 366 |
| #15 | ' Unipolar Depression': ti,ab,kw | 1212 |
| #16 | ' Depression, Unipolar': ti,ab,kw | 1212 |
| #17 | #1 OR #2 OR #3 OR #4 OR #5 OR #6 OR #7 OR #8 OR #9 OR #10 OR #11 OR #12 OR #13 OR #15 OR #16 | 98964 |
| #18 | ' Minocycline': ti,ab,kw | 1271 |
| #19 | 'Minocycline Hydrochloride': ti,ab,kw | 122 |
| #20 | ' Hydrochloride, Minocycline': ti,ab,kw | 122 |
| #21 | ' Minocycline Monohydrochloride ': ti,ab,kw | 0 |
| #22 | 'Apo-Minocycline ': ti,ab,kw | 1 |
| #23 | 'Apo Minocycline ': ti,ab,kw | 2 |
| #24 | #18 OR #19 OR #20 OR #21 OR #22 OR #23 | 1271 |
| #25 | #17 AND #24 | 52 |

Table S2: Inclusion, exclusion criteria, and outcome assessments of the included studies

| Studies | Inclusion criteria | Exclusion criteria | Outcomes measures |
| --- | --- | --- | --- |
| Dean  2017 | Patients with (1) Score above 25 on the MADRS; stable treatment duration for 2 weeks before randomization; (3) female with effective contraception | (1) Discontinued medication for 7 consecutive days; (2) became pregnant or withdrew consent. (3) discontinued with severe adverse events | (1) change of MADRS score;(2) change of Clinical Global Impression scale (CGI); (3) change of Patient Global Impression (PGI) scale; (4) Quality of life (Q-LES-Q-SF); |
| Husain  2017 | Patients (1) aged 18 to 65 years; (2) consent to participate; (3) stable treatment duration for 4-6 weeks before randomization; (4) failed to remit with at least two courses of antidepressant treatment (5) able to take oral medication and (6) female with effective contraception | (1) relevant medical illness (renal, hepatic, cardiac, serious dermatological disorders such as exfoliative dermatitis, systemic lupus erythematosus); (2) history of intolerance to tetracyclines; (3) concomitant penicillin therapy; (4)concomitant anticoagulant therapy; (5) presence of a seizure disorder; (6) currently taking valproic acid; (7) any change of psychotropic medications within the previous 4 weeks; (8) diagnosis of substance-use disorder (except nicotine or caffeine) or dependence within the last 3 months according to DSM-5 criteria; (9) pregnant or breast-feeding or (10) presence of primary psychotic disorder. | (1) HAMD-17 score; (2) Response (reduction of 50% or more of the HAMD-17); (3) Remission (score of ≤7 of the HAMD-17); (3) CGI scale; (4) Patient Health Questionnaire (PHQ-9); (5) quality of life (EQ-5D) |
| Nettis  2021 | Patients (1) aged 25–60; (2) non-responders to stable treatment duration for 6 weeks; (3)HAMD-17＞14; (3) accepting augmentation with minocycline;(4) CRP levels ≥1 mg/L;(5) no changes in current therapy. | (1) active suicidal ideation; (2) diagnosed with bipolar disorder, obsessive compulsive disorder, eating disorder, post-traumatic stress disorder, or substance/alcohol misuse disorder; (3) taking warfarin; (4) having a history of sensitivity or intolerance to tetracycline; (5) having an autoimmune or inflammatory disorder; (6) having hepatic or renal failure; (7) taking other medicine. | (1) change of HAMD-17 score; (2) Response (reduction of 50% of the HAMD-17) and partial response (reduction of 25% of the HAMD-17); (3) changes of inflammatory biomarkers; (4)change of BDI scale; (5)change of CGI scale |
| Emadi-kouchak  2016 | HIV patients (1) aged between 18 and 55 years; (2) diagnosed with mild to moderated depression according to the DSM-IV-TR criteria; (3)Hamilton Depression Rating Scale (HDRS) score up to 18 | (1) severe depression and suicidal ideation; (2) taking antidepressants in the last month; (3) allergy to minocycline or other tetracyclines;(4) cancer, pregnancy, and any cognitive disorders including dementia due to HIV. | (1) change of HAMD-17 score |
| Hellmann-Regen  2022 | Patients (1) aged between 18 and 75 years, (2) HAMD-17 up to 16; (3) inadequate response to stable treatment duration for 6 weeks | NA | (1) change of MADRS scale;(2) response (50% reduction in MADRS); (3) remission (MADRS score <9); (4) change of BDI scale;(5) change of CGI scale; (6) Symptom Checklist 90-R (SCL-90-R) (7) inflammation parameters |

Table S3: Risk of quality assessment (based on GRADE scale)

|  | Within-study bias | Reporting bias | Indirectness | Heterogeneity | Incoherence | Confidence rating | Reasons for downgrading |
| --- | --- | --- | --- | --- | --- | --- | --- |
| Husin 2017 | No concern | Low risk | No concern | Low risk | Low risk | High | [NA] |
| Dean 2017 | No concern | Low risk | No concern | Low risk | Low risk | High | [NA] |
| Emadi-Kouchak 2016 | No concern | Low risk | No concern | Low risk | Some concerns | Moderate | ["Incoherence"] |
| Nettis 2021 | No concern | Low risk | No concern | Low risk | Low risk | High | [NA] |
| Hellmann-Regen 2022 | No concern | Low risk | No concern | Low risk | Low risk | High | [NA] |


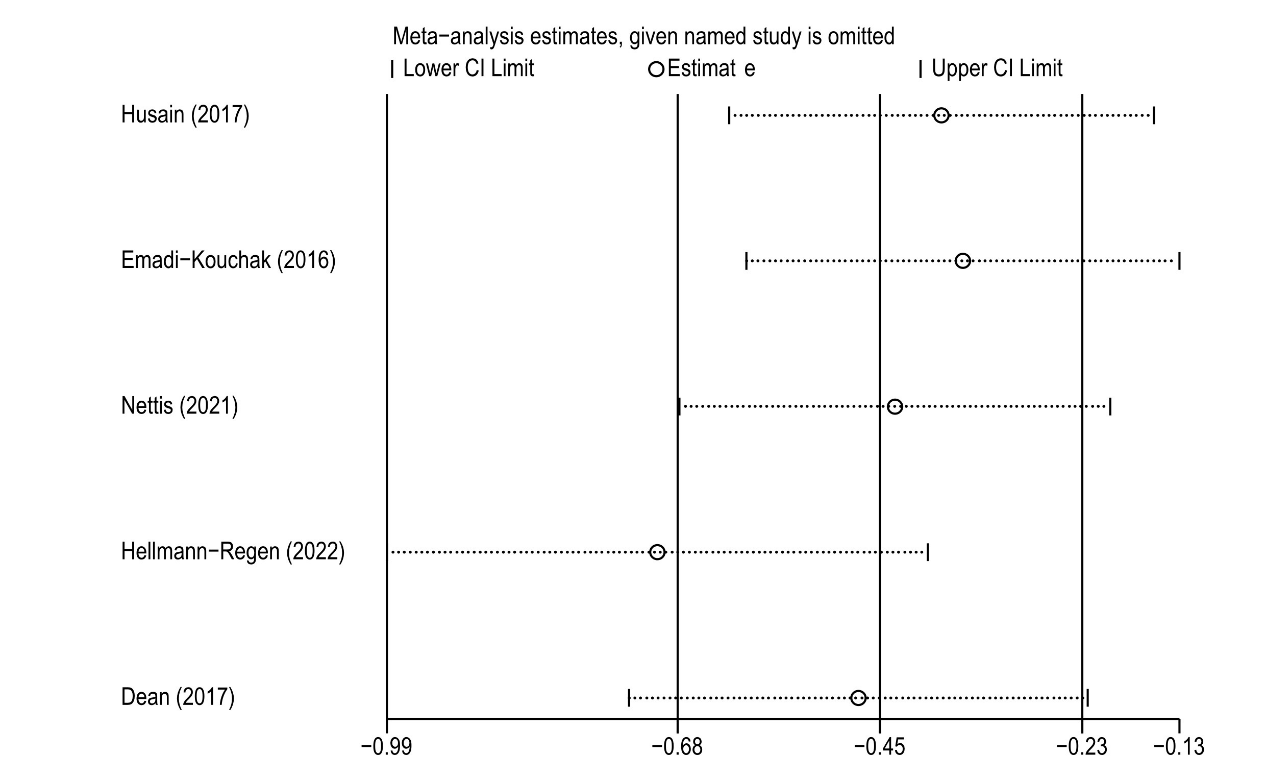


Figure S1. Sensitivity analysis for changes in depressive severity score


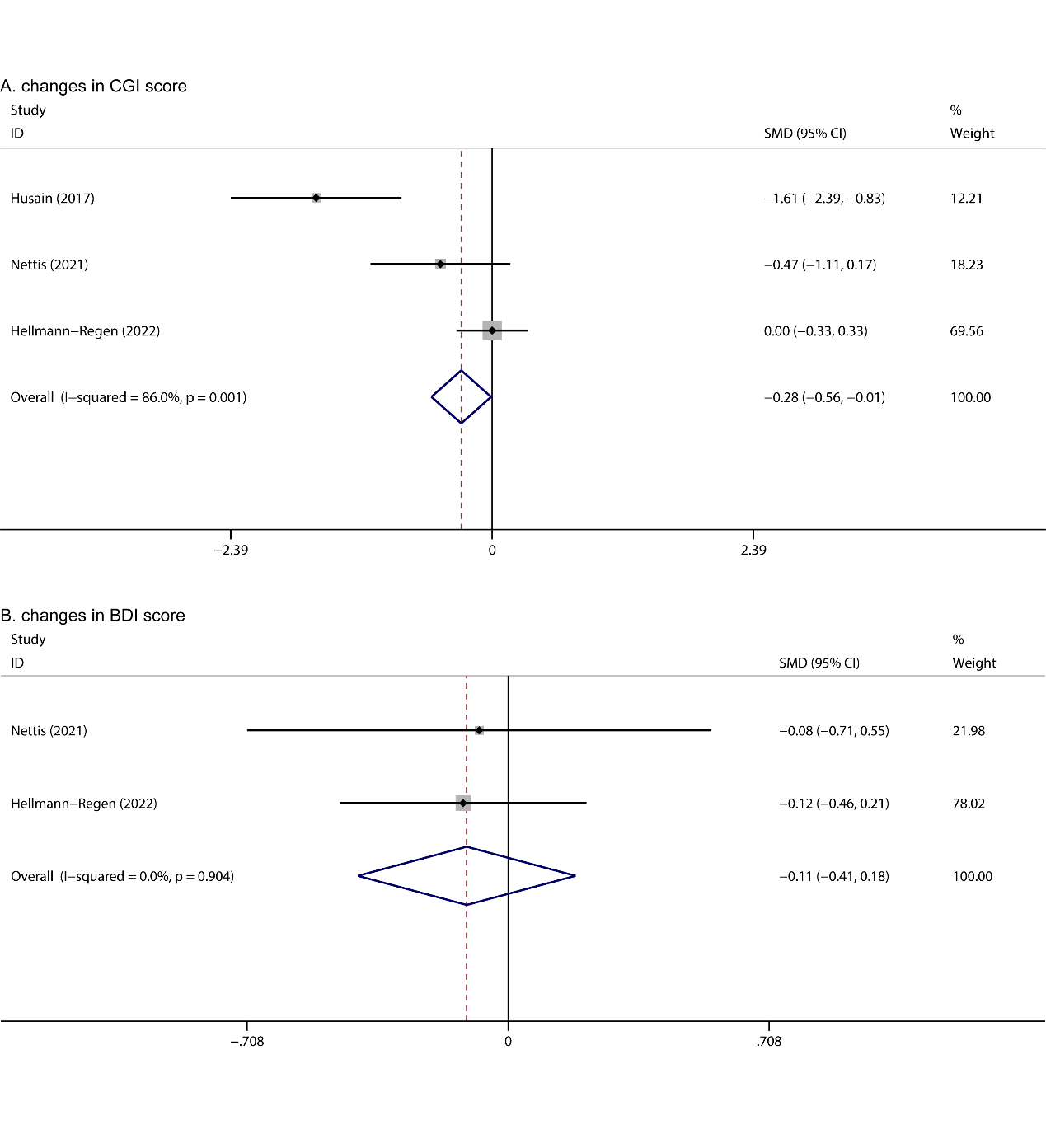


Figure S2. Forest plot for changes in (A) CGI and (B) BDI score


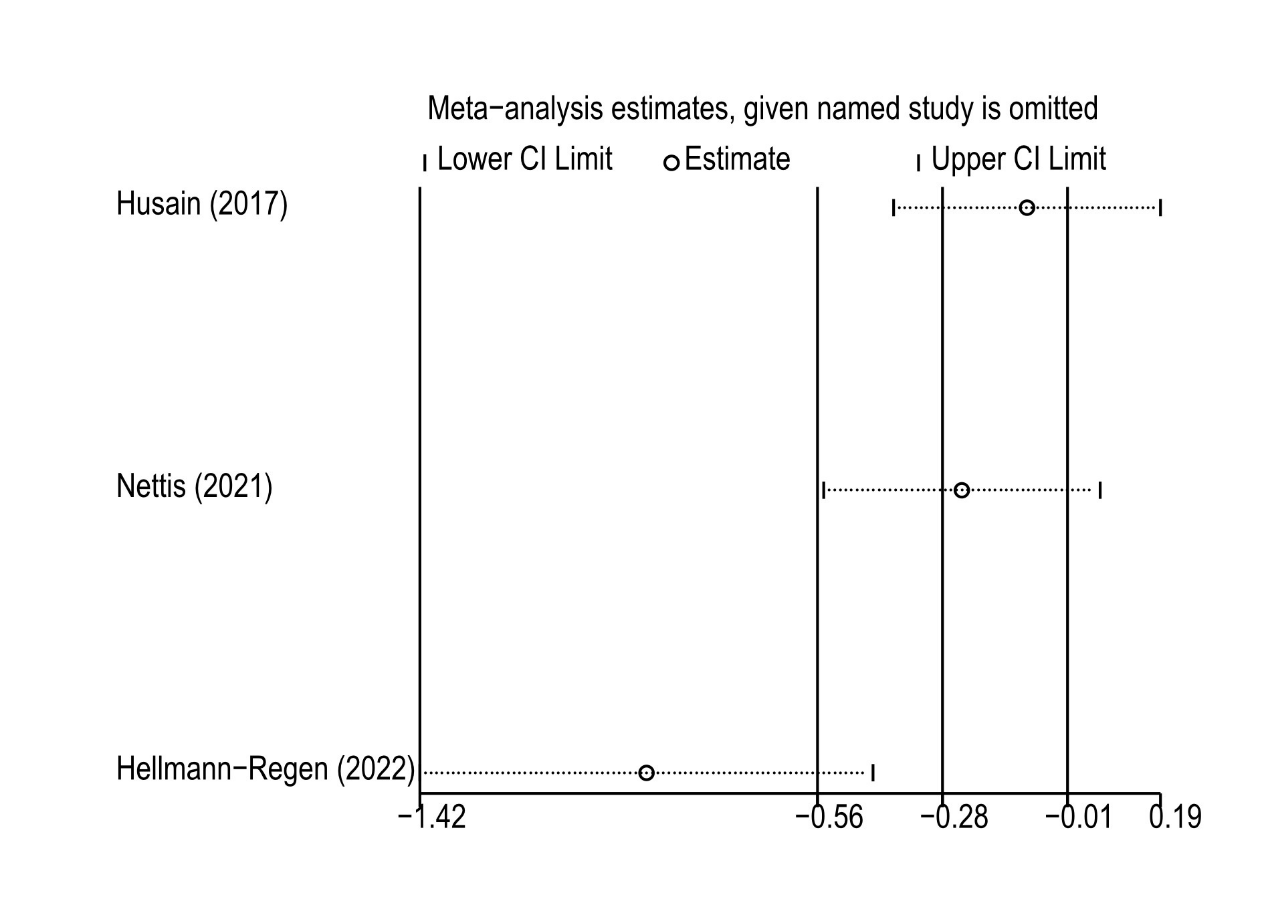


Figure S3. Sensitivity analysis for change of CGI score


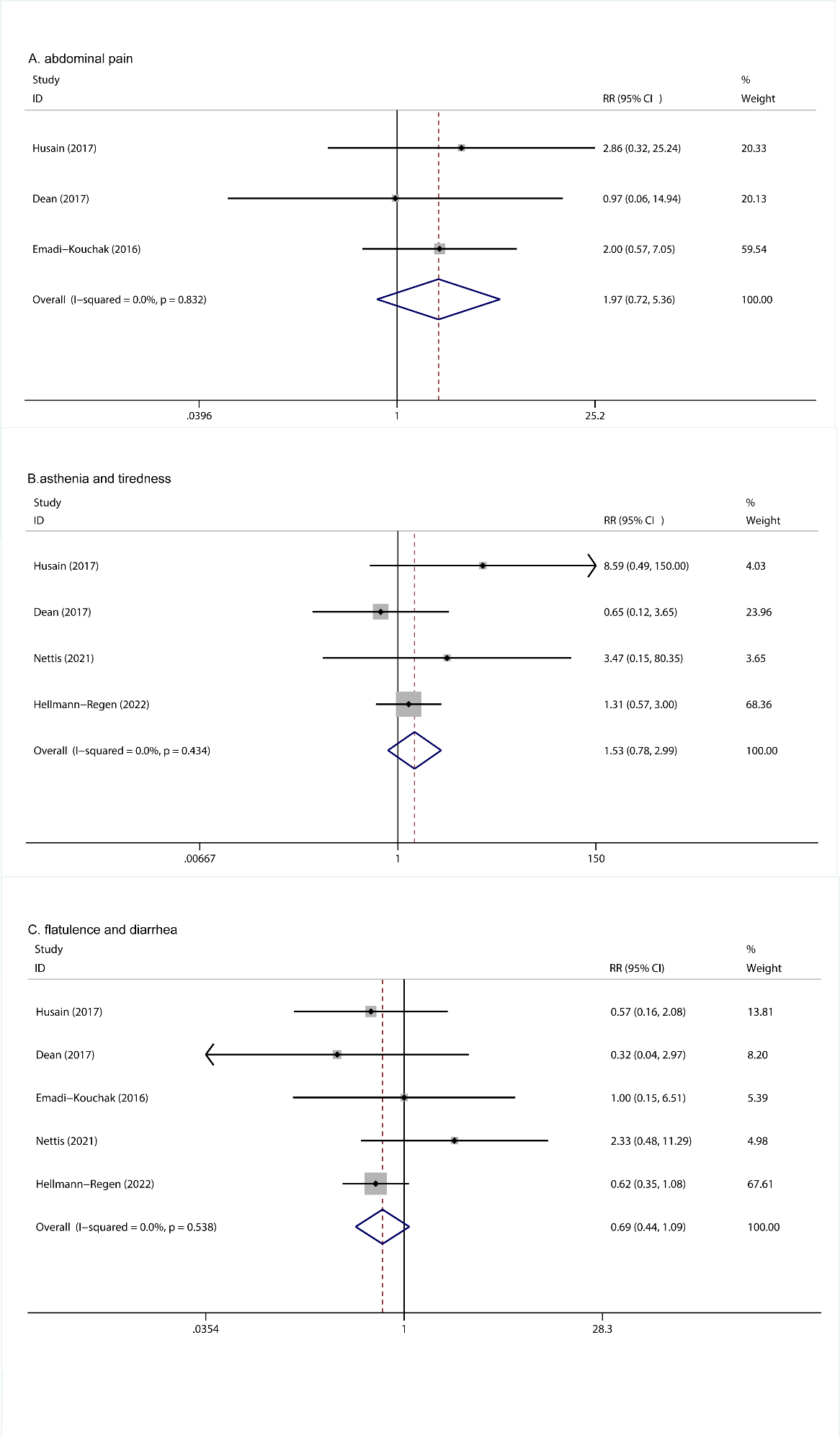
 Figure S4. Forest plot for (A) abdominal pain; (B) asthenia and tiredness; (C) Flatulence and diarrhea


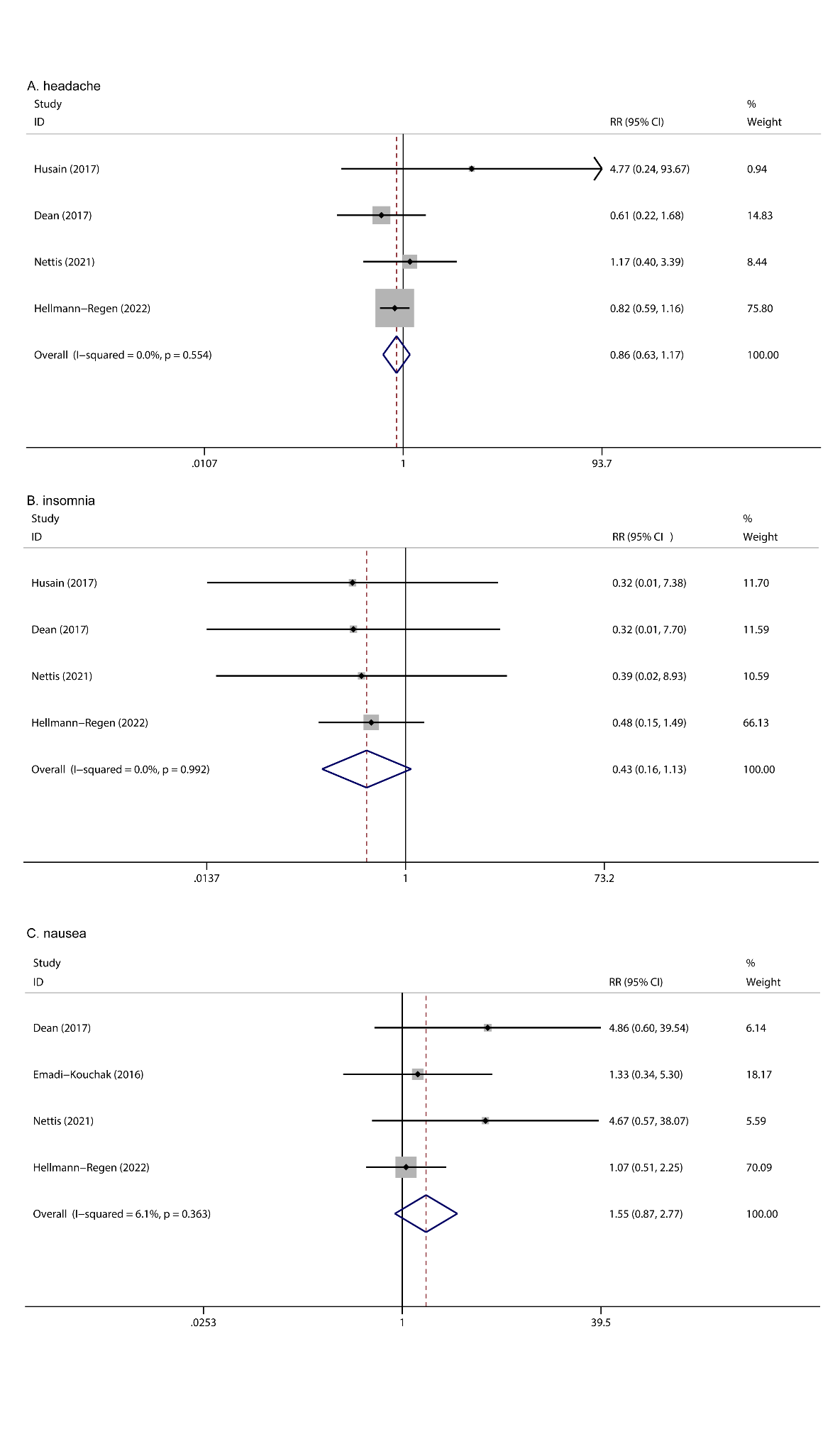


Figure S5. Forest plot for (A) headache; (B) insomnia; (C) nausea


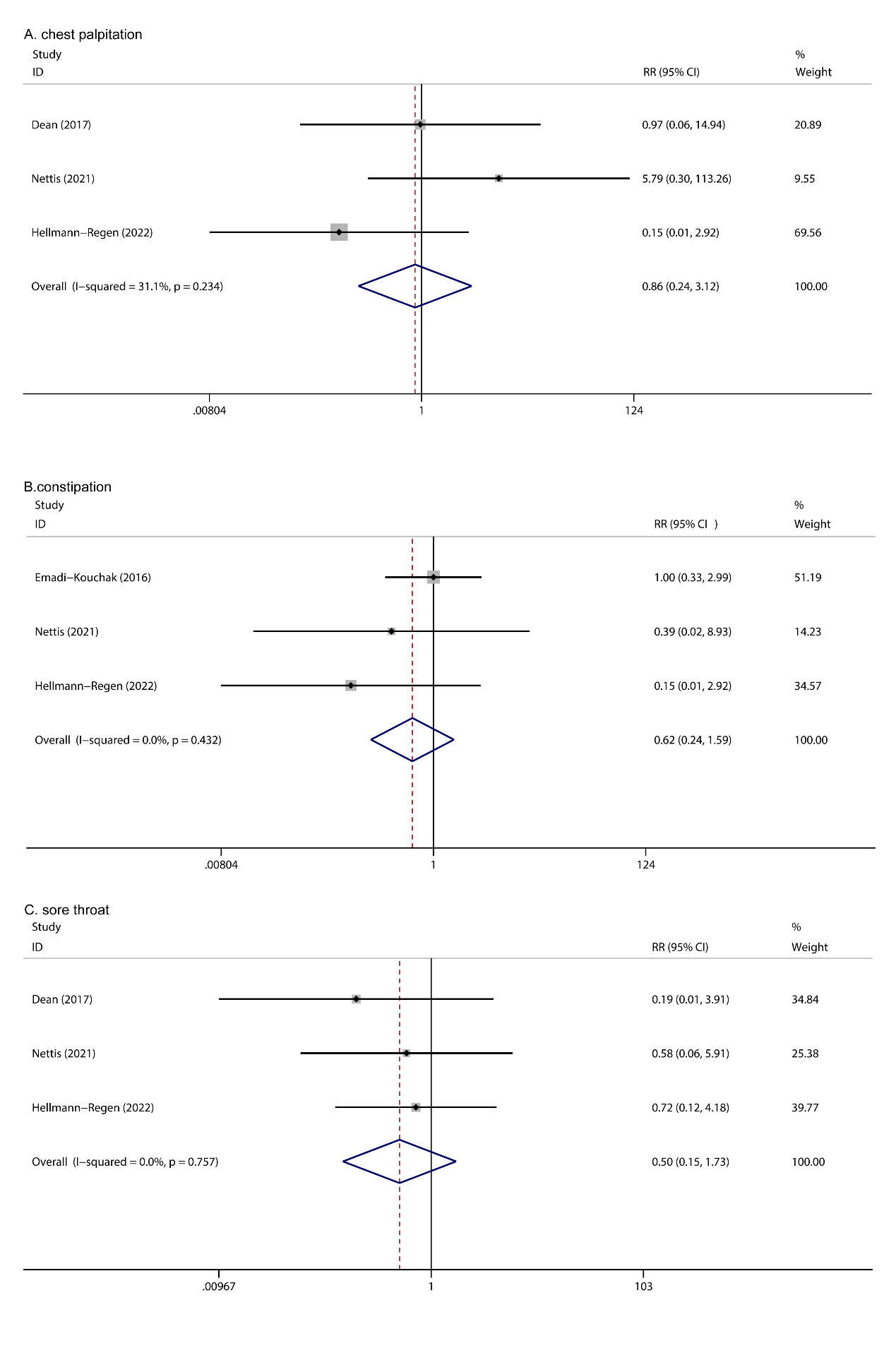


Figure S6. Forest plot for (A) chest palpitation; (B) constipation; (C) sore throat


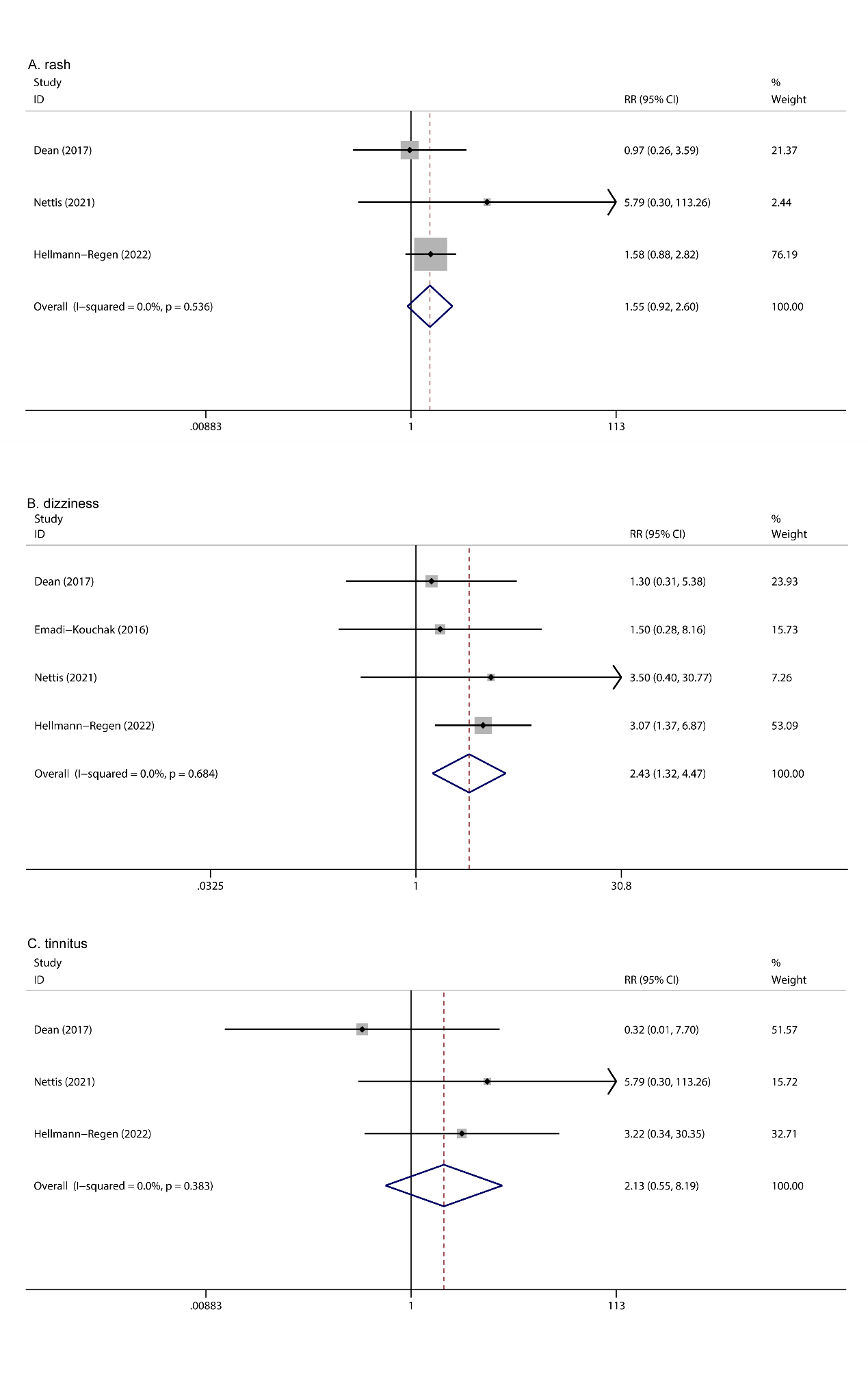


Figure S7. Forest plot for (A) rush; (B) dizziness; (C) tinnitus


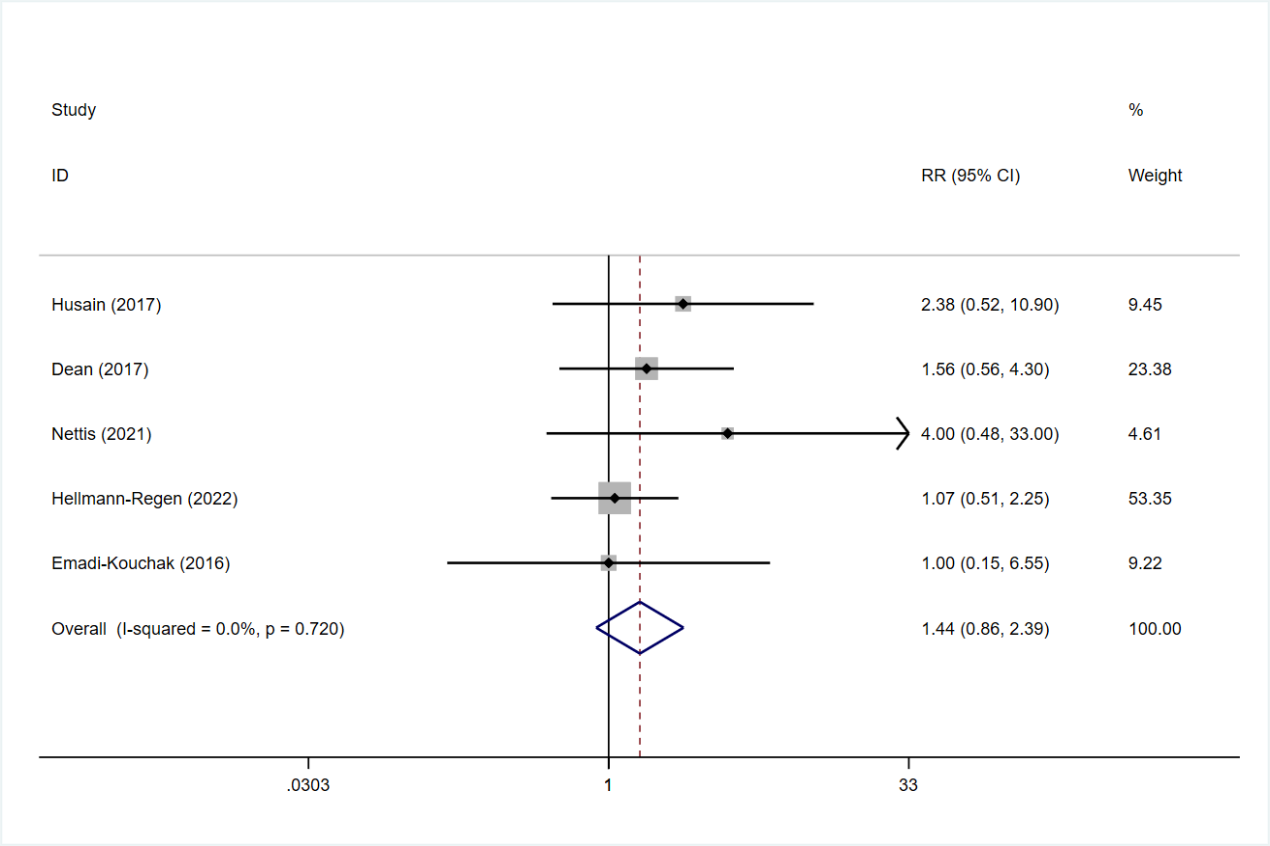


Figure S8. Forest plot for all-cause discontinuation


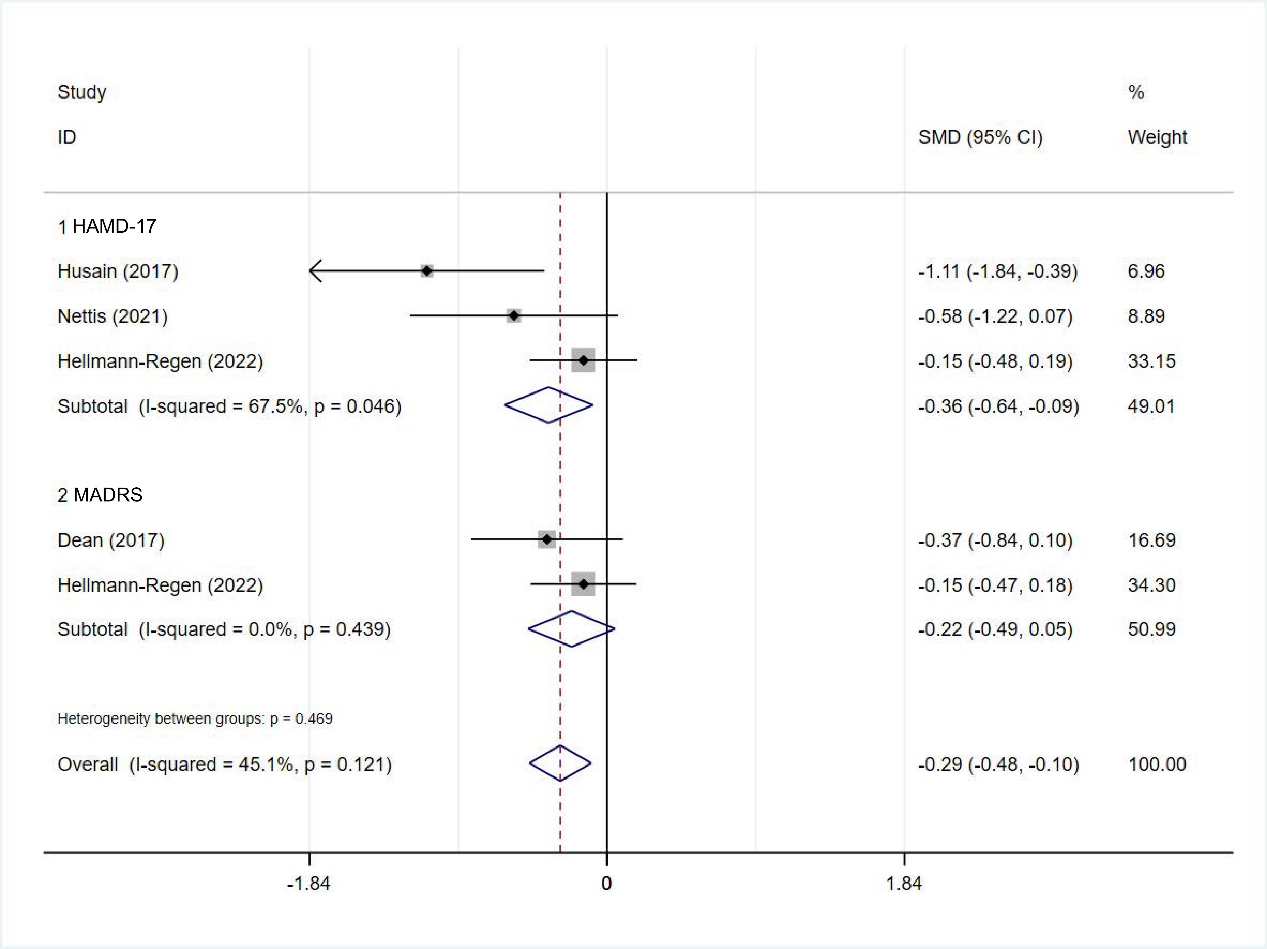


Figure S9. Forest plot for subgroup analysis of (A) HAMD-17 and (B) MADRS in patients with MDD


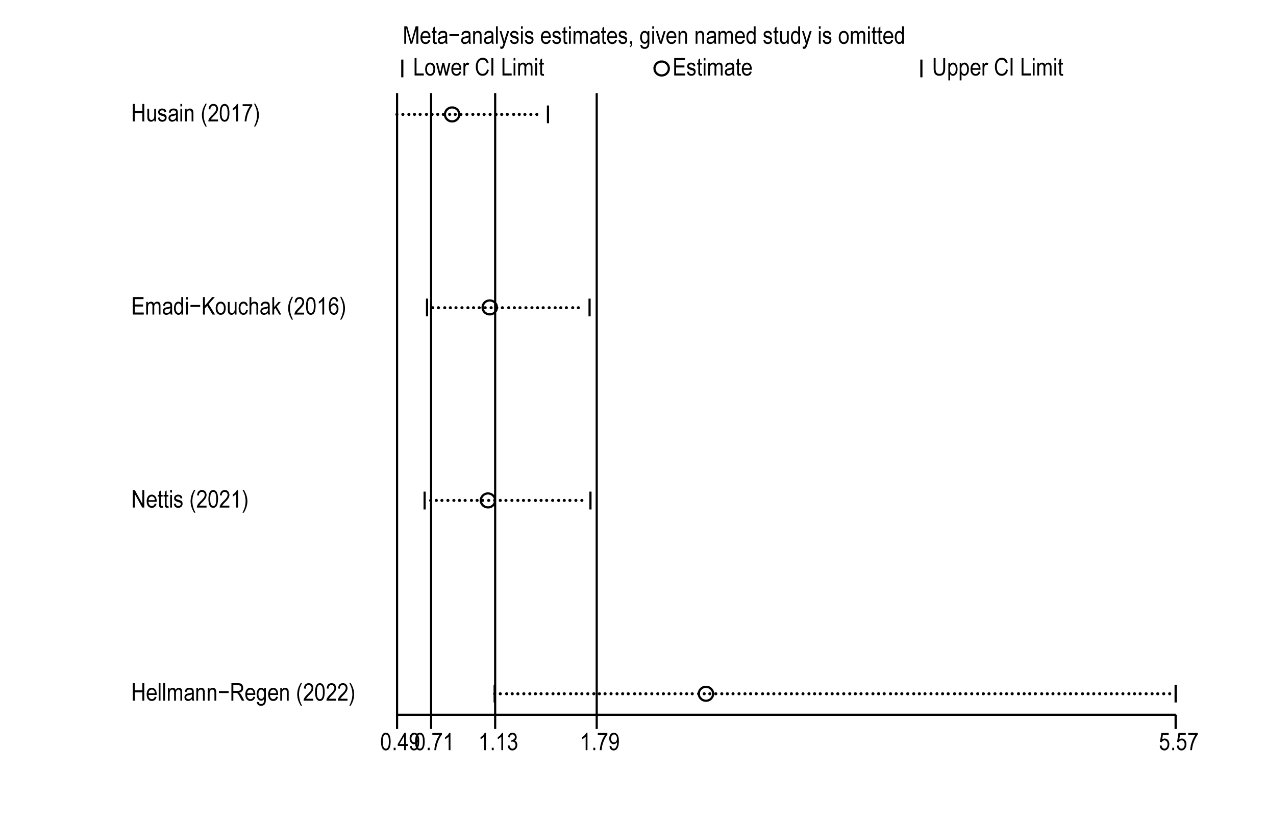


Figure S10. Sensitivity analysis for response in depressive patients


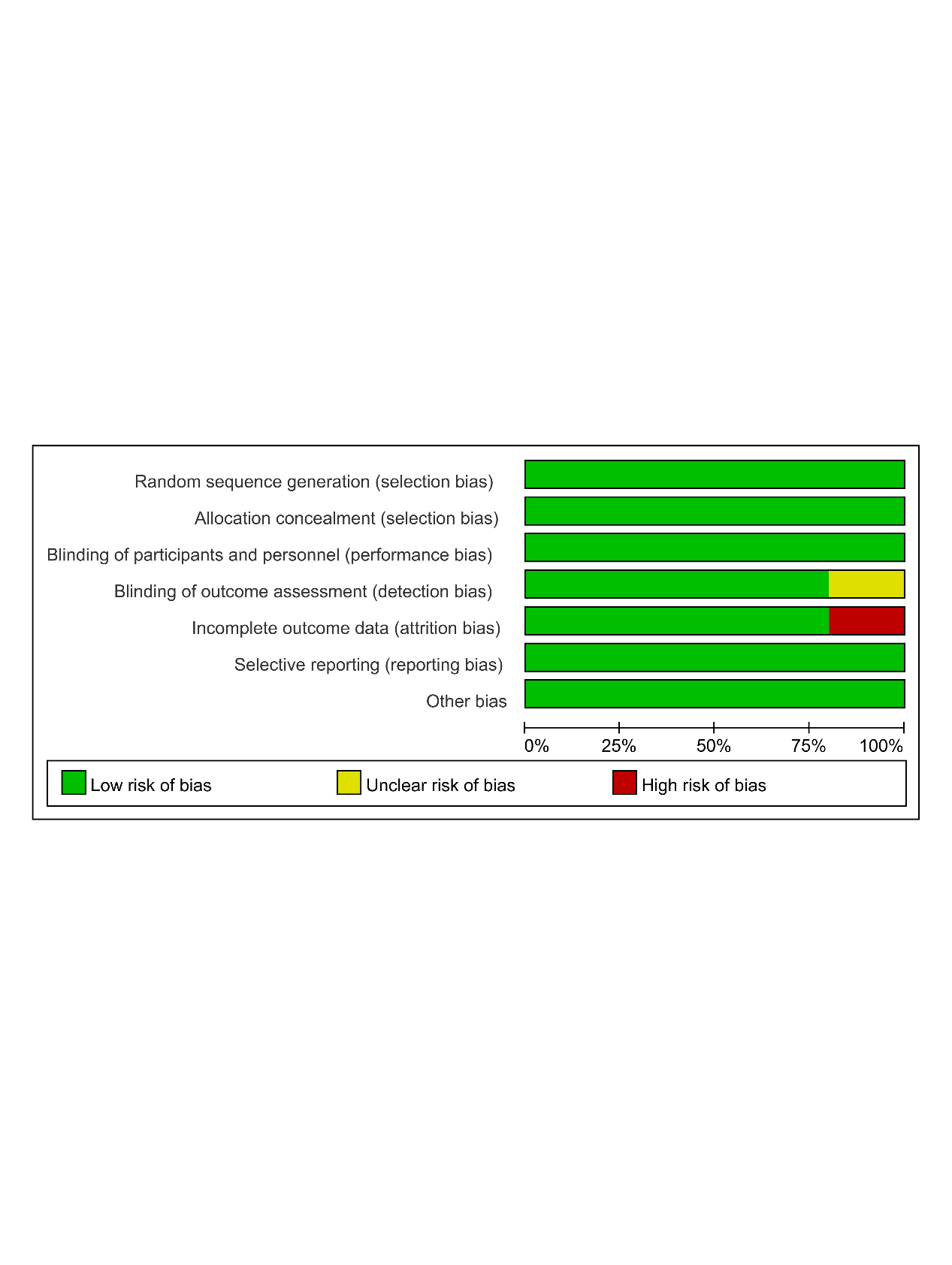


Figure S11: The overall risk of bias


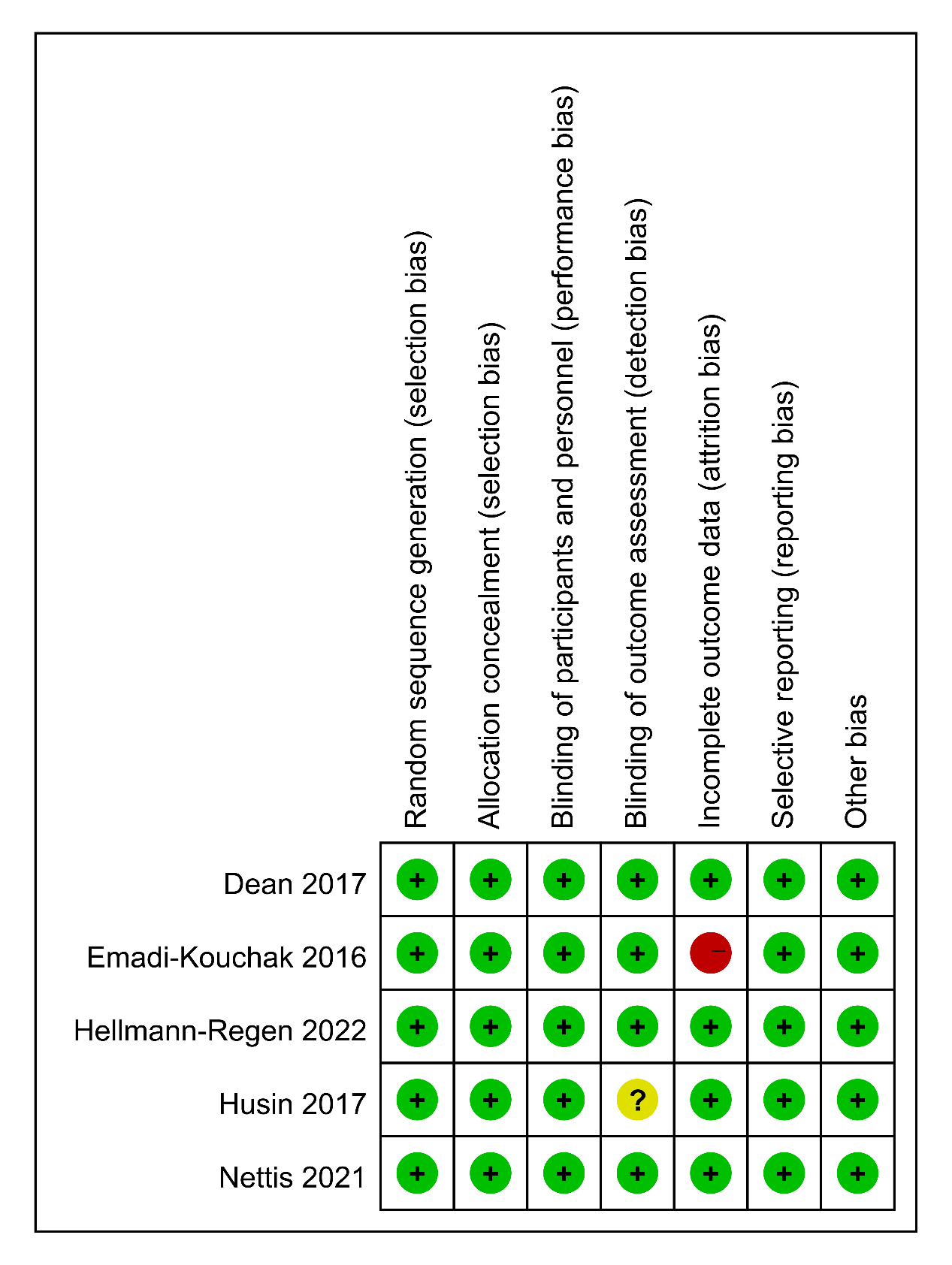


Figure S12: The individual risk of bias for each study
